# Supplementary material for: Efficient Access to 3,5-Disubstituted 7-(Trifluoromethyl)pyrazolo[1,5-a]pyrimidines Involving SNAr and Suzuki Cross-Coupling Reactions
Source: Molecules. 2020 Apr 28;25(9):2062. doi: 10.3390/molecules25092062 (PMC7248703; doi:10.3390/molecules25092062)

molecules

MDPI

Article

Efficient Access to 3,5-Disubstituted 7-(Trifluoromethyl) pyrazolo[1,5-*a*]pyrimidines involving S_NAr and Suzuki Cross-Coupling Reactions

Badr Jismy [1], Tikad Abdellatif [2], Mohamed Akssira [3], Gérald Guillaumet [4] and Mohamed Abarbri [1,*]

[1] Laboratoire de Physico-Chimie des Matériaux et des Electrolytes pour l'Energie (PCM2E), EA 6299, Avenue Monge Faculté des Sciences, Parc de Grandmont, 37200 Tours, France; badr.jismy@hotmail.com (B.J.); mohamed.abarbri@univ-tours.fr (M.A.).

[2] Laboratoire de Chimie Moléculaire et Substances Naturelles, Faculté des Sciences, Université Moulay Ismail, B.P. 11201, Zitoune, Meknès 50050, Morocco; abdel.tikad@gmail.com.

[3] Laboratoire de Chimie Physique & de Chimie Bioorganique, URAC 22, Université Hassan II de Casablanca, BP 146, 28800 Mohammedia, Morocco; akssira.m@gmail.com.

[4] Institut de Chimie Organique et Analytique (ICOA), Université d'Orléans, UMR CNRS 7311, BP 6759, Rue de Chartres, 45067 Orléans Cedex 2, France; gerald.guillaumet@univ-orleans.fr.

* Correspondence: mohamed.abarbri@univ-tours.fr; Tel.: +33(2)47 36 73 59; Fax : +33(2)47 36 70 73.

3-Bromo-7-(trifluoromethyl)pyrazolo[1,5-a]pyrimidin-5-one (**4**)

^{1}H NMR (300 MHz, DMSO-d_6)

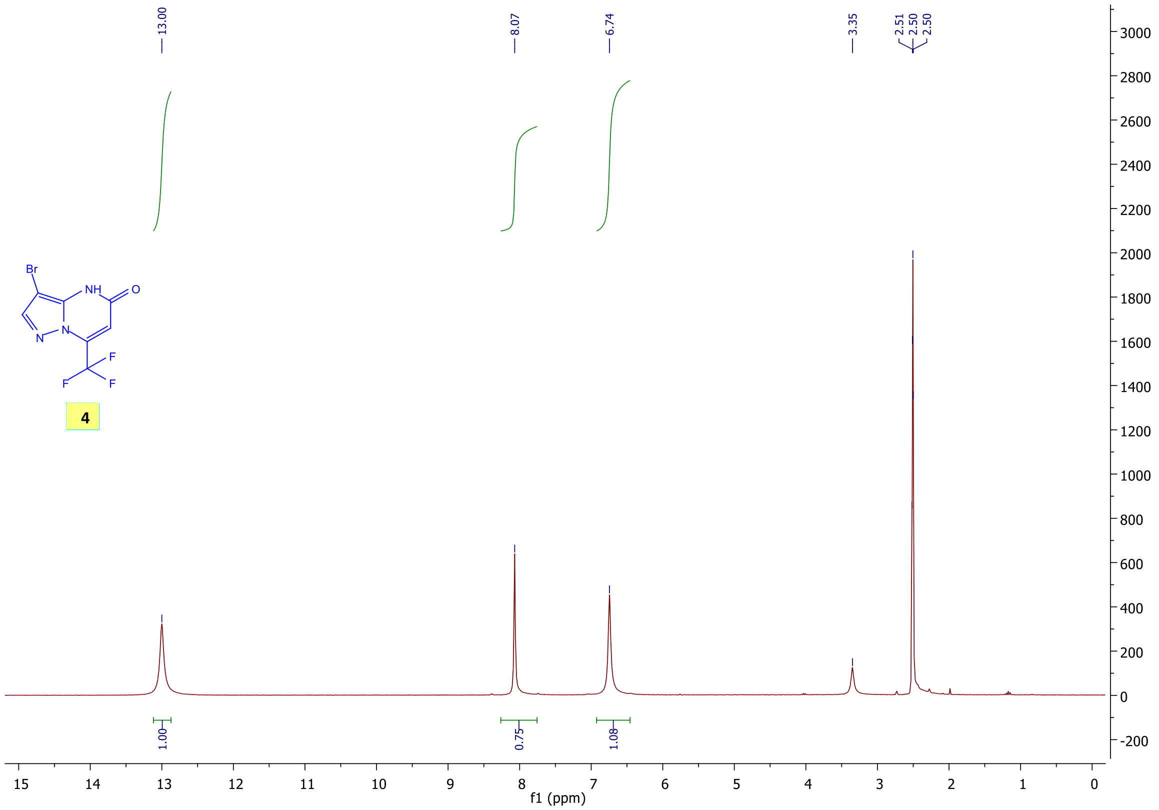

^{19}F NMR (282 MHz, DMSO-d_6)

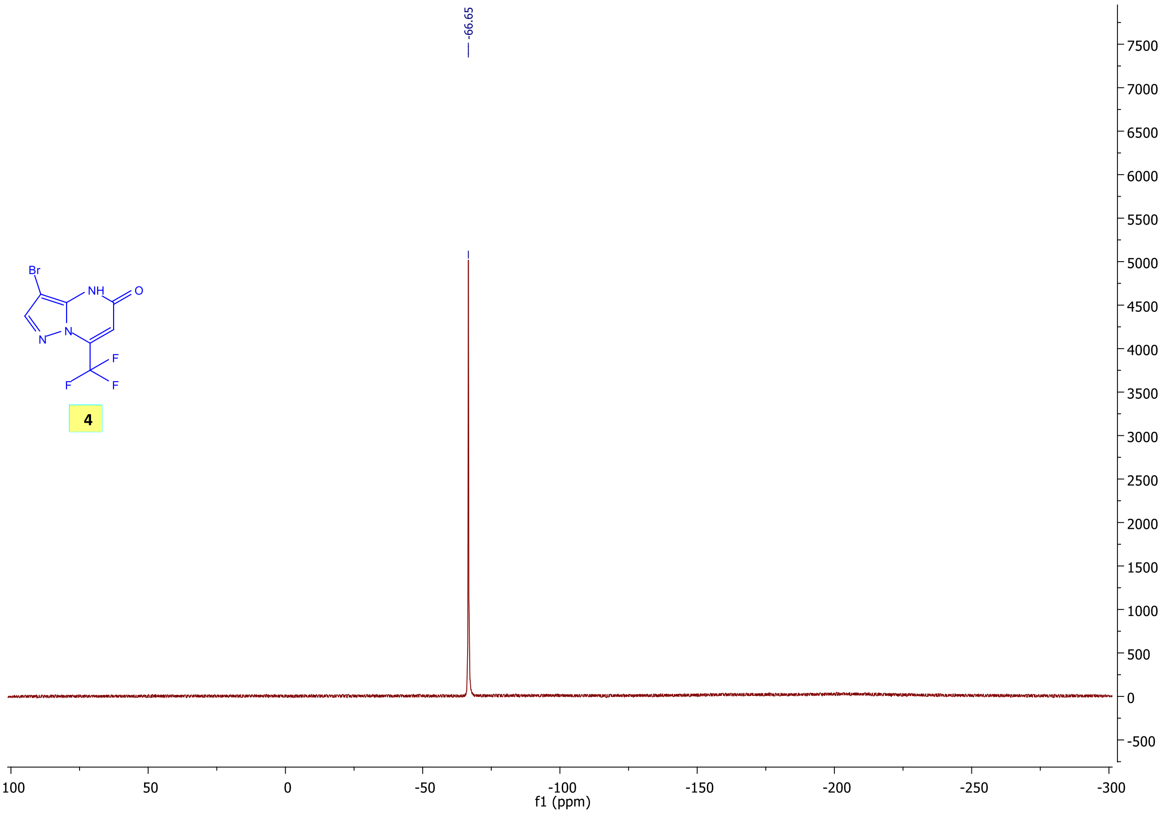

^{13}C NMR (75 MHz, DMSO-d_6)

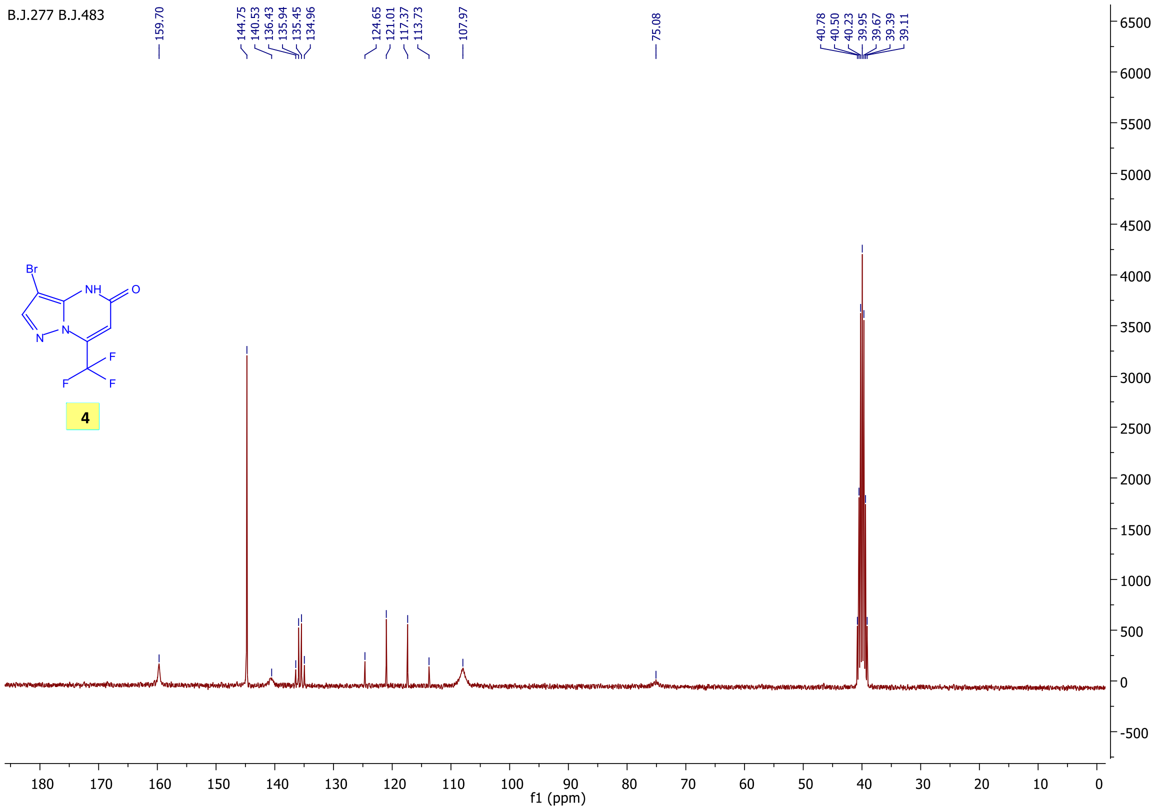

3-Bromo-5-[N-(4-methoxybenzyl)amino]-7-(trifluoromethyl)pyrazolo[1,5-a]pyrimidine (**5a**)

^{1}H NMR (300 MHz, $CDCl_3$)

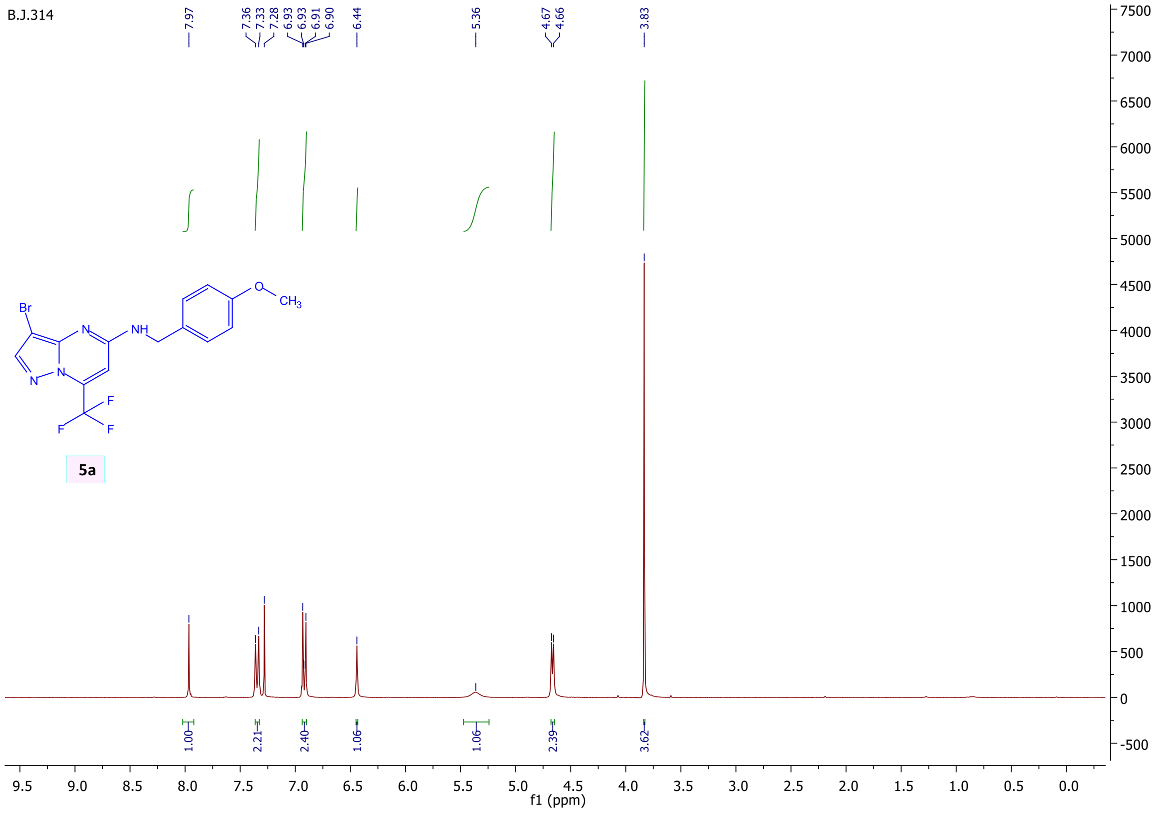

^{19}F NMR (282 MHz, $CDCl_3$)

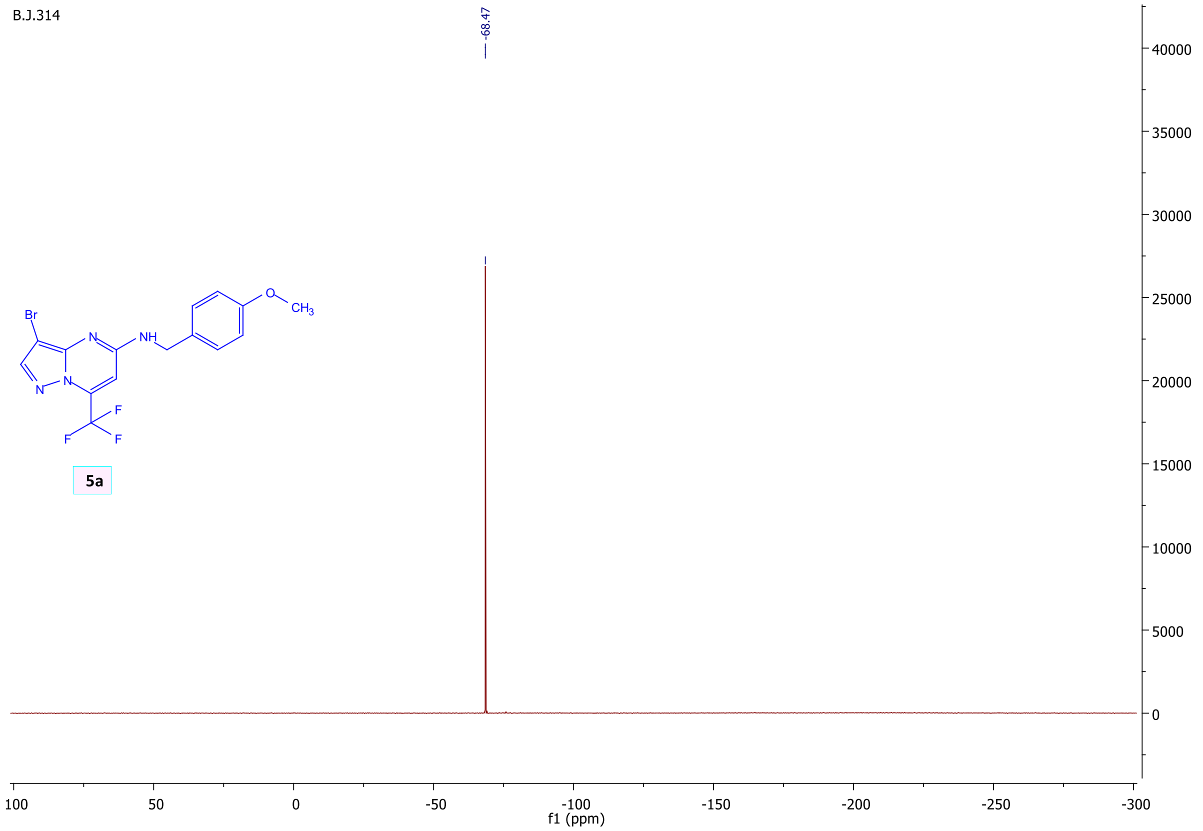

^{13}C NMR (75 MHz, Acétone-d_6)

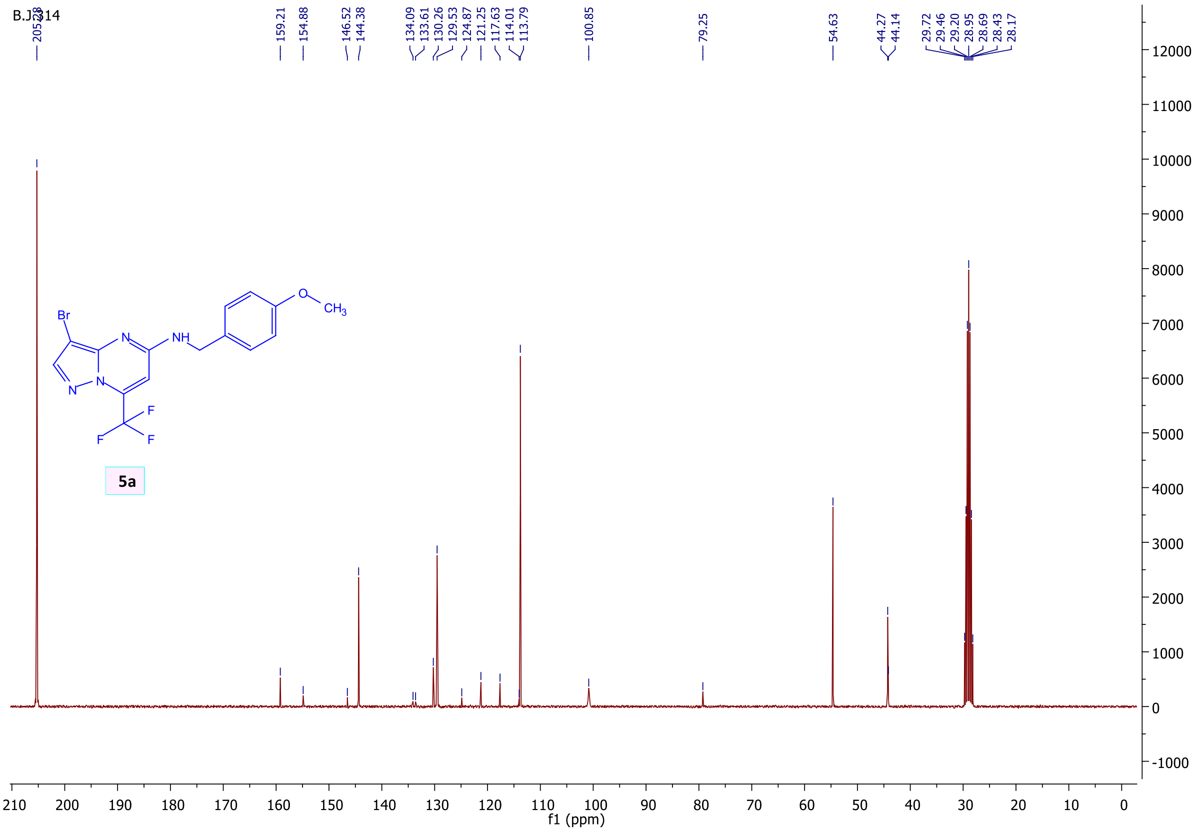

5-(N-benzylamino)-3-bromo-7-(trifluoromethyl)pyrazolo[1,5-a]pyrimidine (**5b**)

^{1}H NMR (300 MHz, $CDCl_3$)

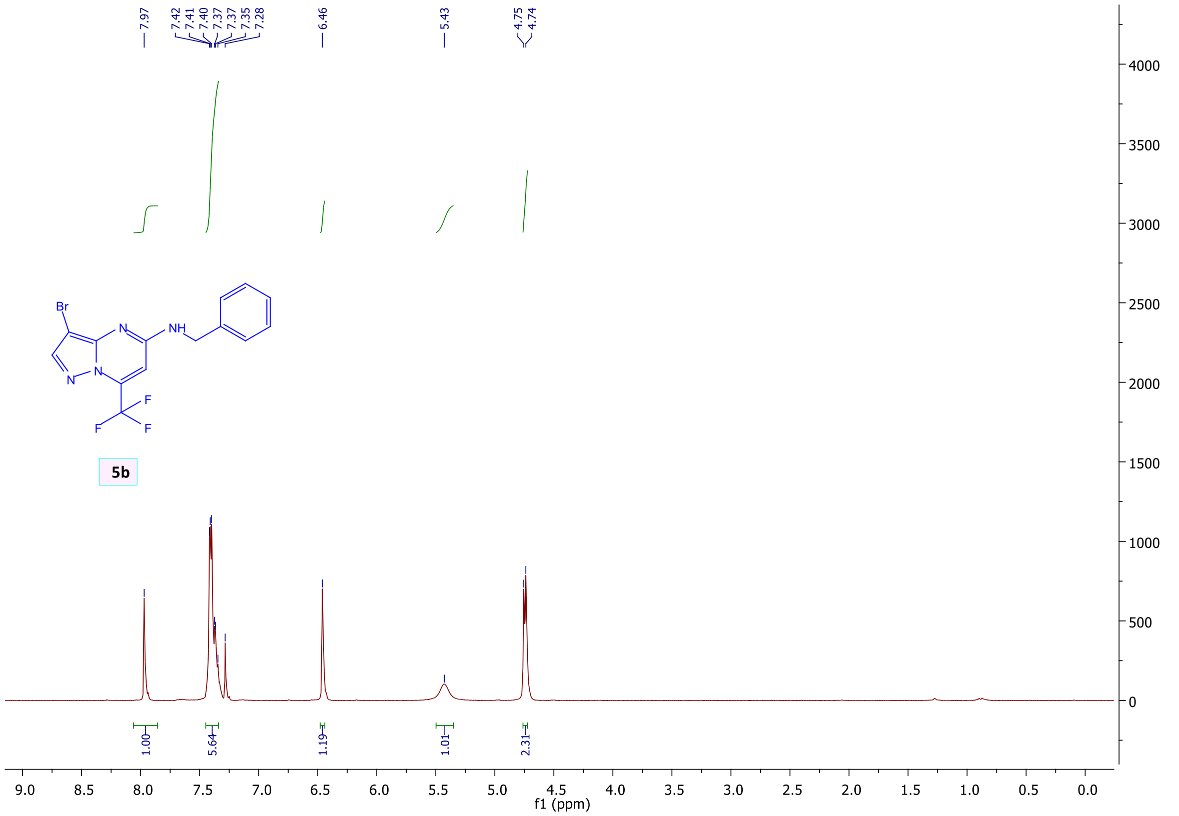

^{19}F NMR (282 MHz, $CDCl_3$)

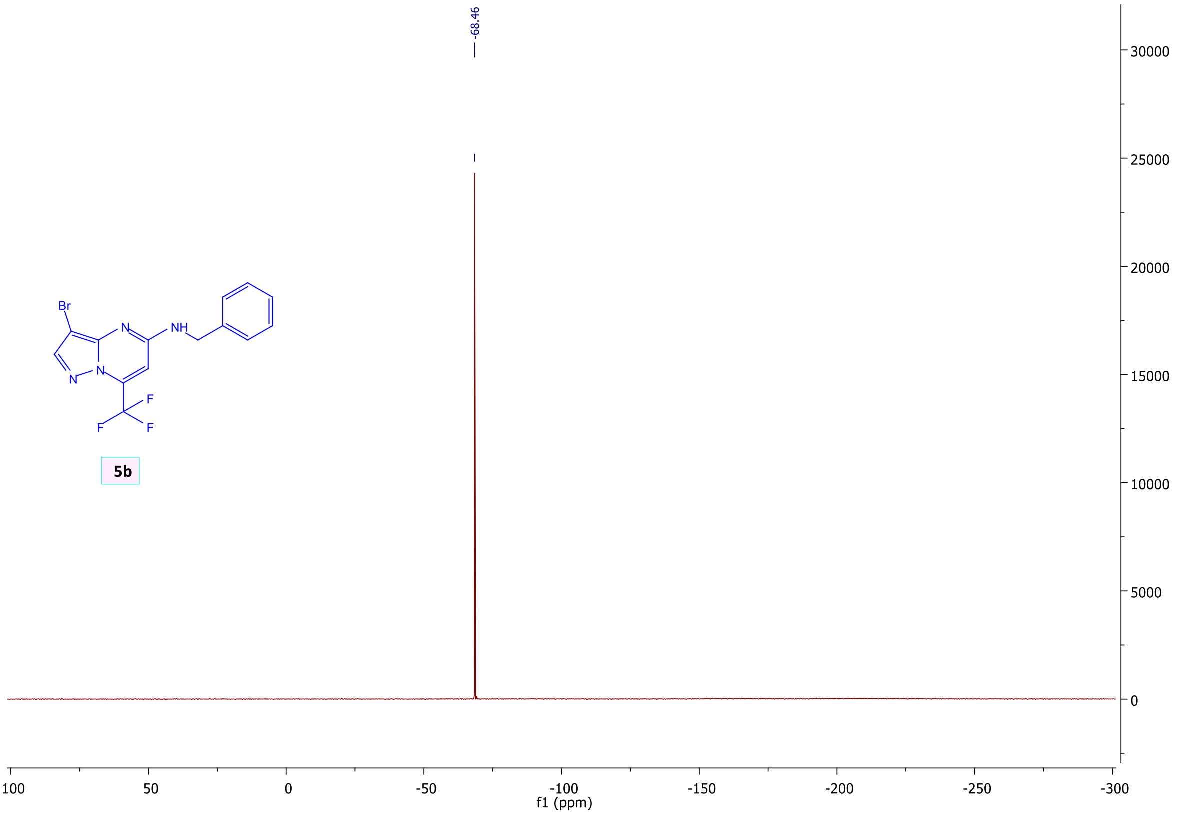

^{13}C NMR (75 MHz, Acétone-*d*$_6$)

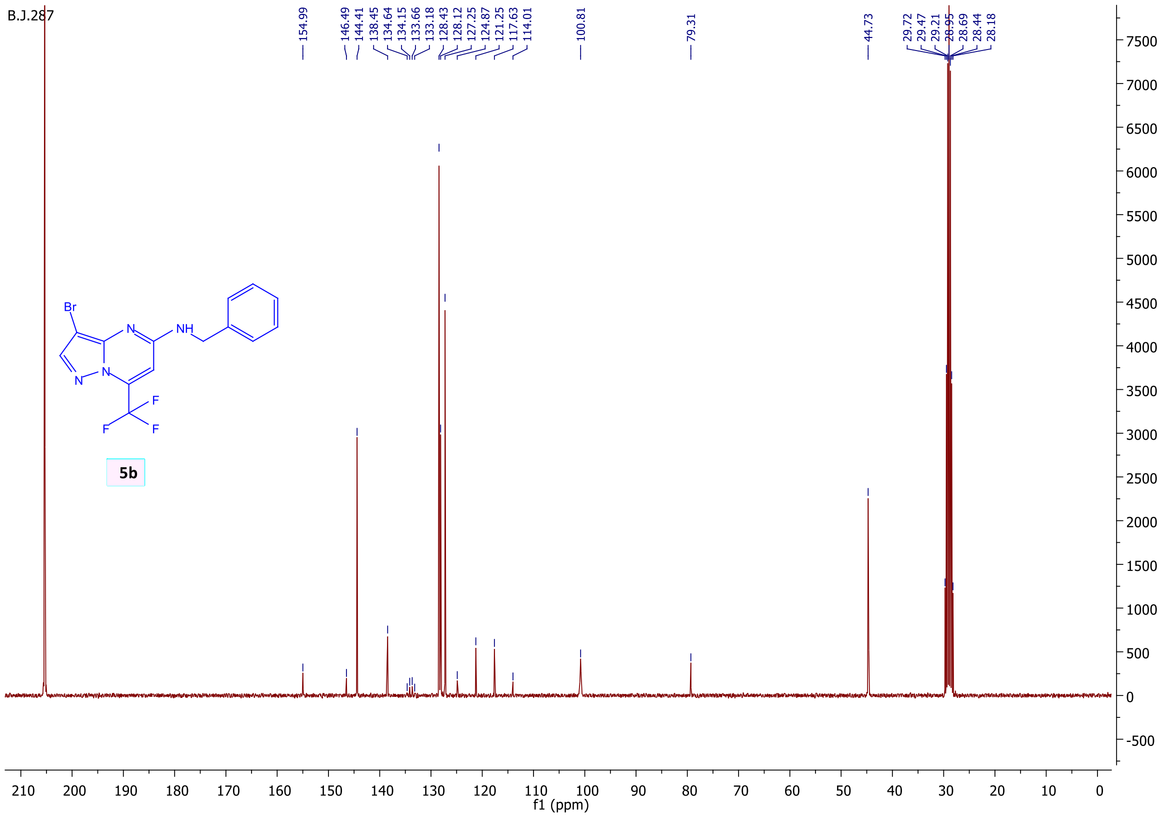

3-bromo-5-[2-(tertiobutoxycarboxylamino)-N-éthylamino]-7-(trifluoromethyl)pyrazolo[1,5-a]pyrimidine (**5c**)

^{1}H NMR (300 MHz, $CDCl_3$)

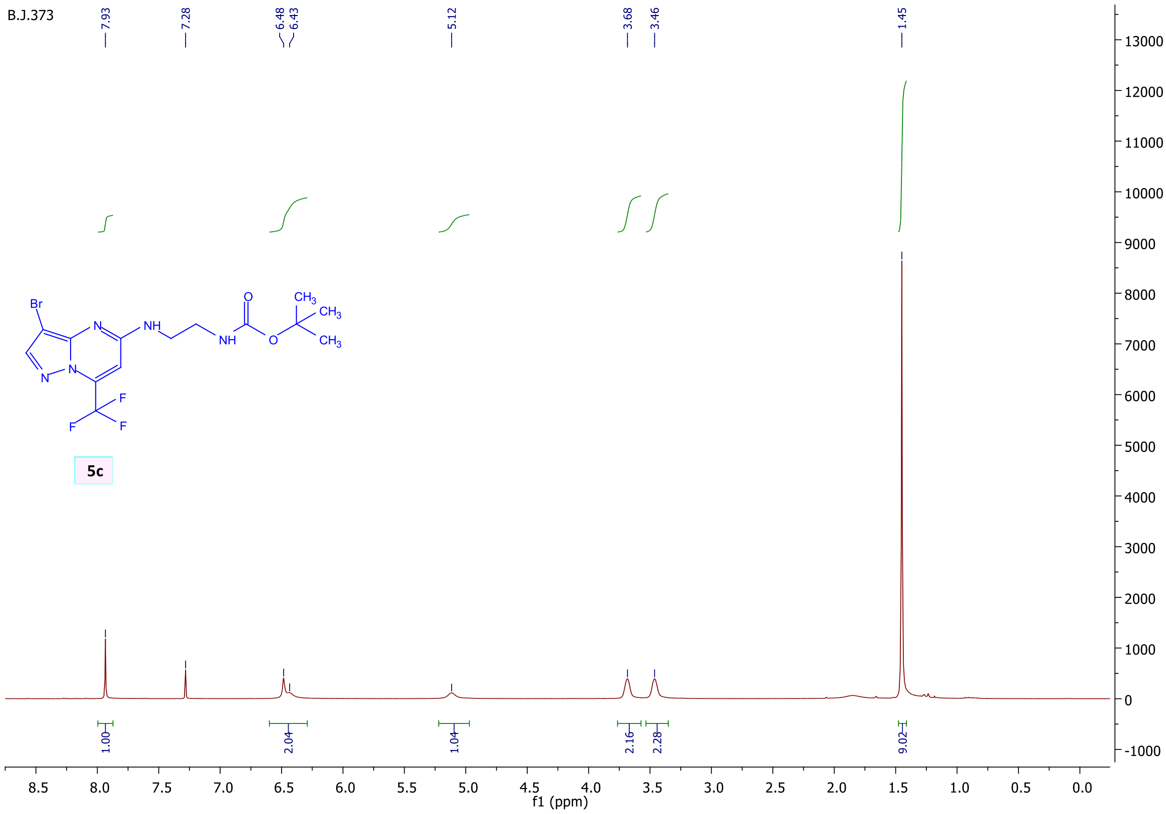

^{19}F NMR (282 MHz, $CDCl_3$)

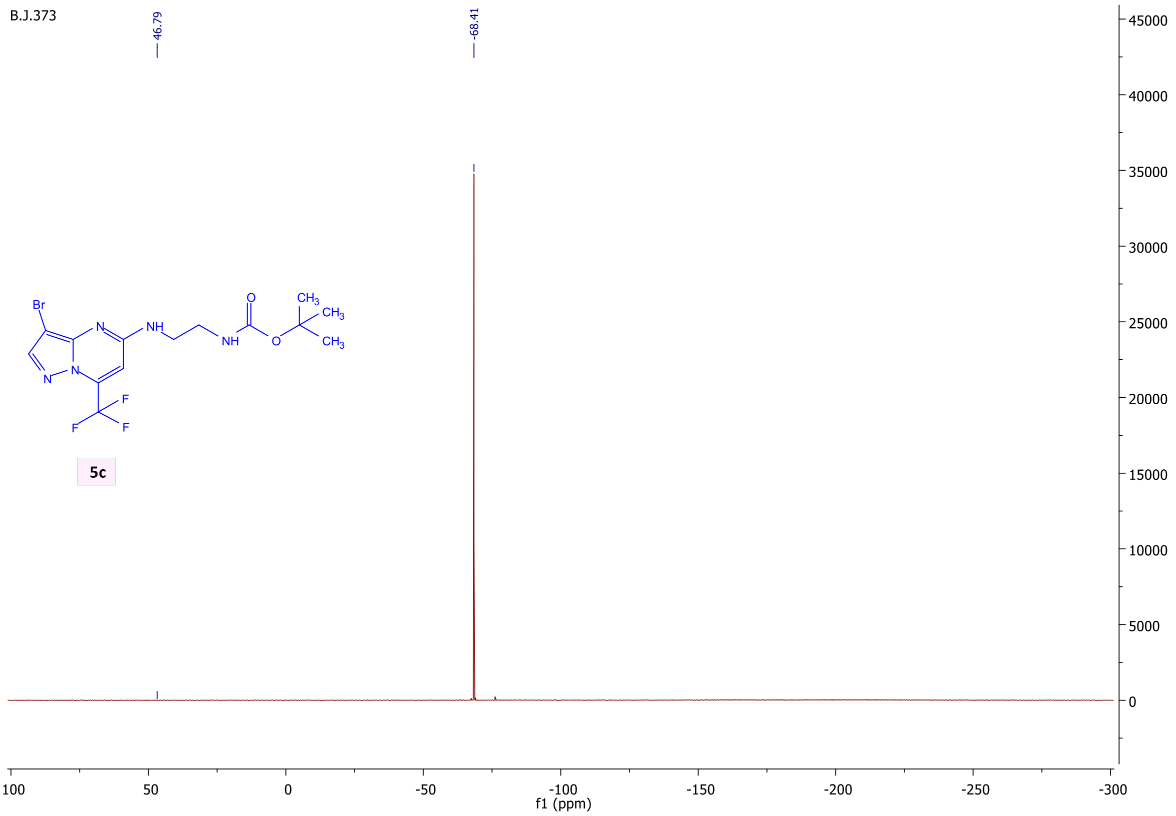

^{13}C NMR (75 MHz, Acétone-*d*$_6$)

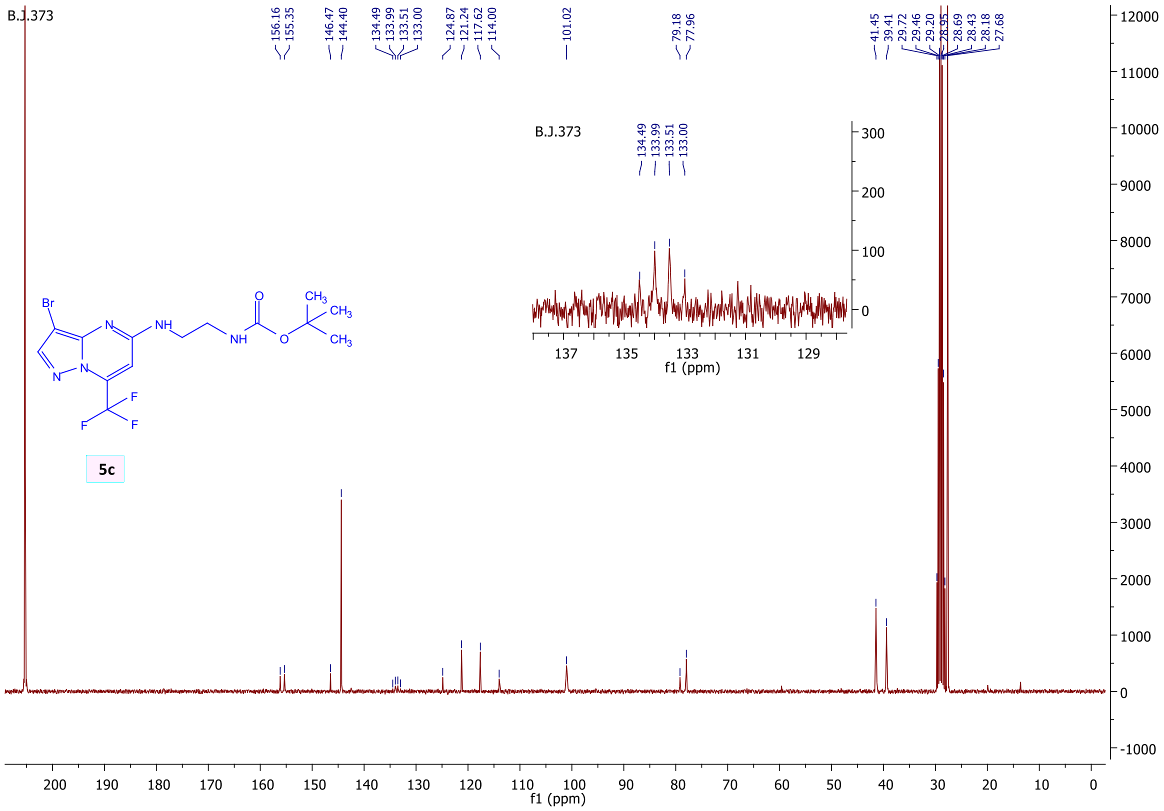

3-bromo-5-N-[4-(hydroxycyclohexyl)amino]-7-(trifluoromethyl)pyrazolo[1,5-a]pyrimidine (**5d**)

^{1}H NMR (300 MHz, Acétone-*d*₆)

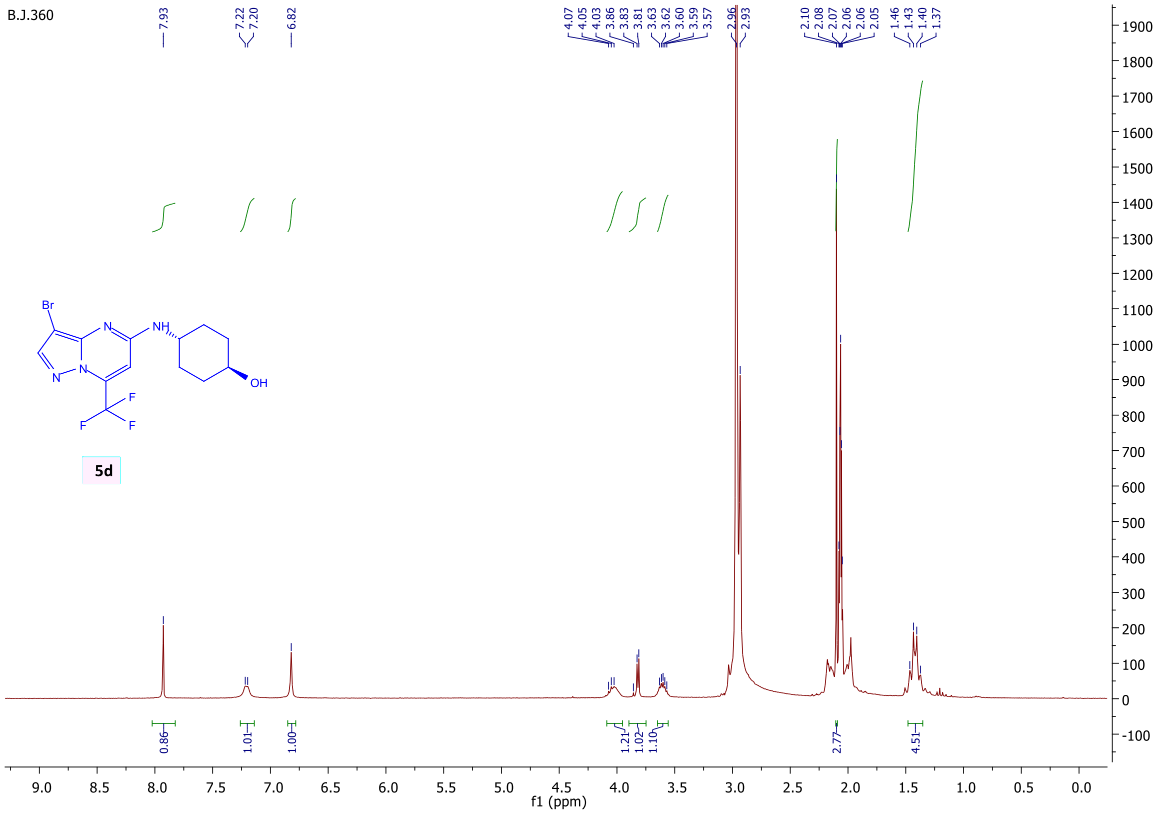

^{19}F NMR (282 MHz, Acétone-*d*₆)

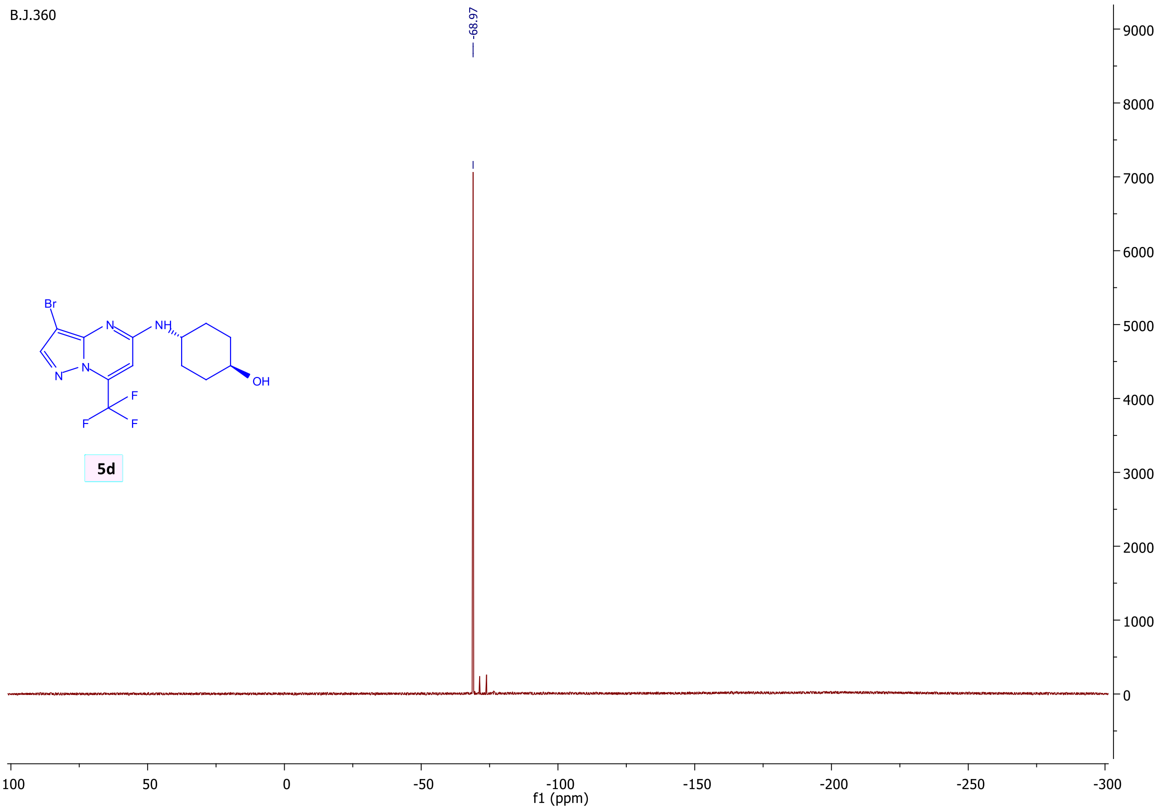

^{13}C NMR (75 MHz, Acétone-d_6)

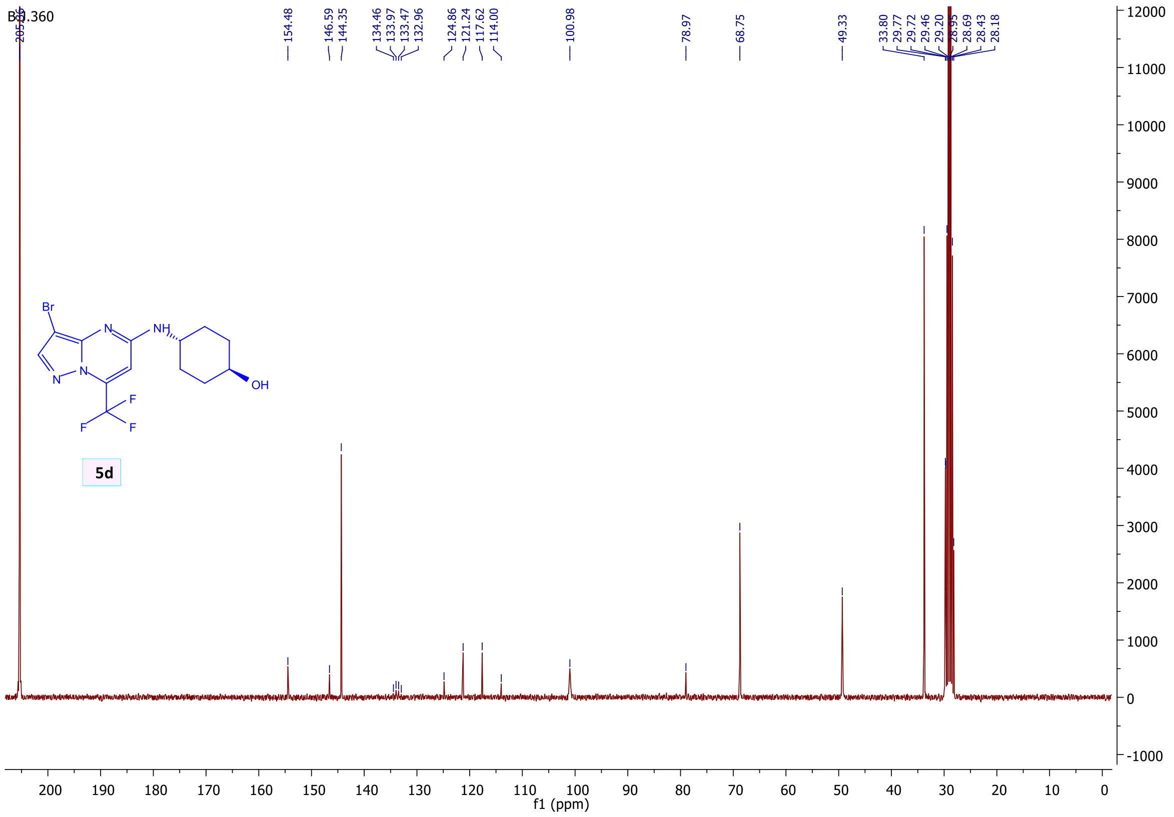

3-bromo-N-[(prop-2-ynyl)amino]-7-(trifluoromethyl)pyrazolo[1,5-a]pyrimidine (**5e**)

^{1}H NMR (300 MHz, CDCl$_3$)

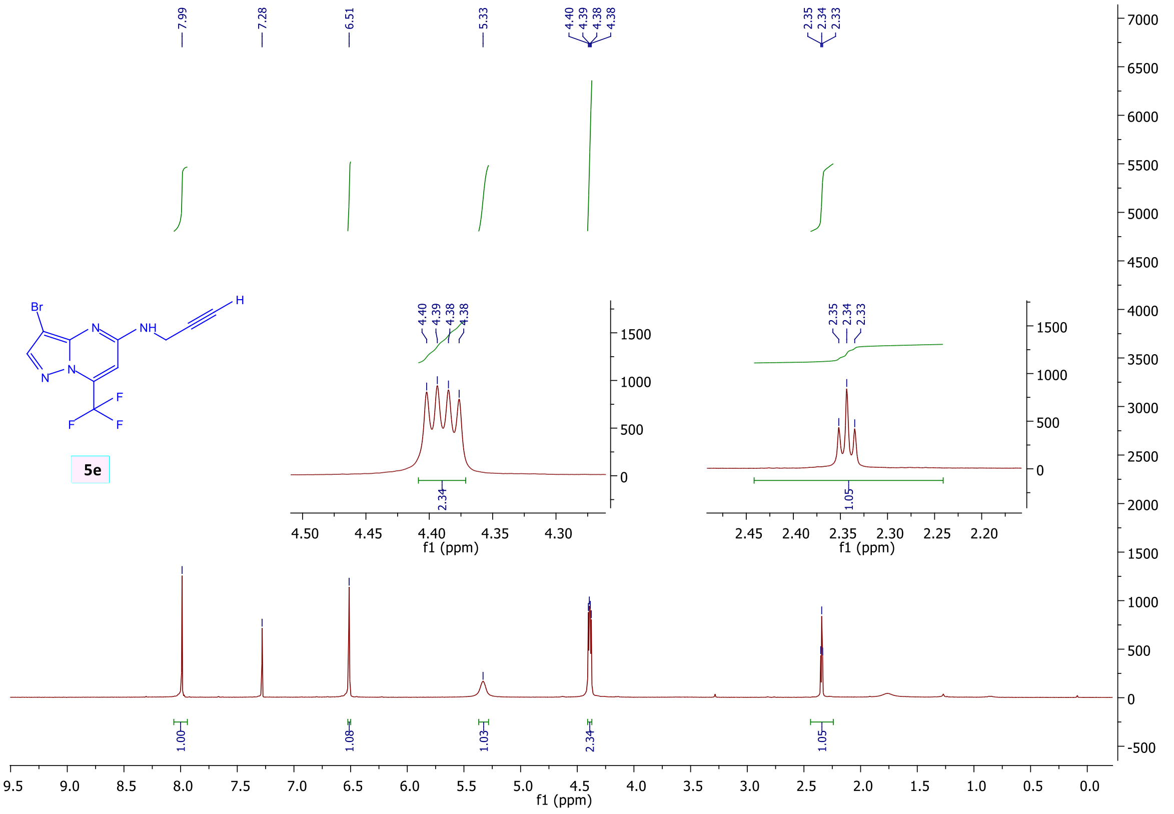

^{19}F NMR (282 MHz, $CDCl_3$)

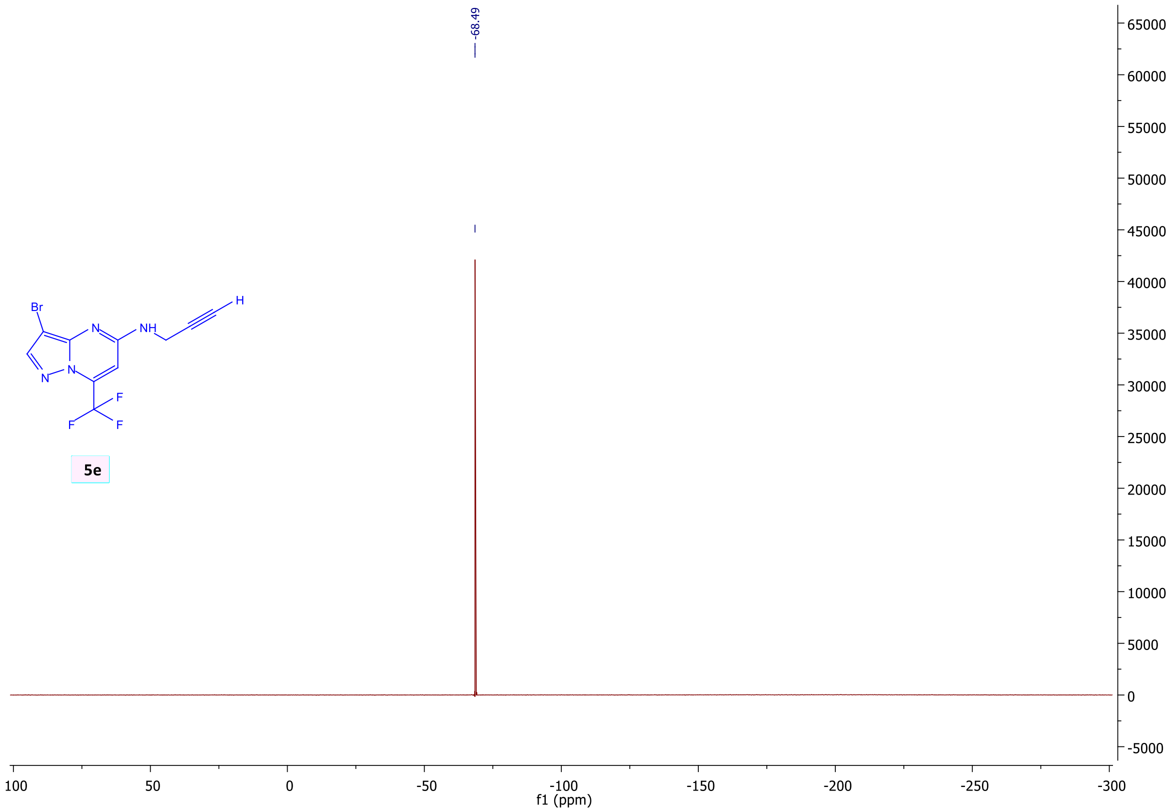

^{13}C NMR (75 MHz, Acétone-d_6)

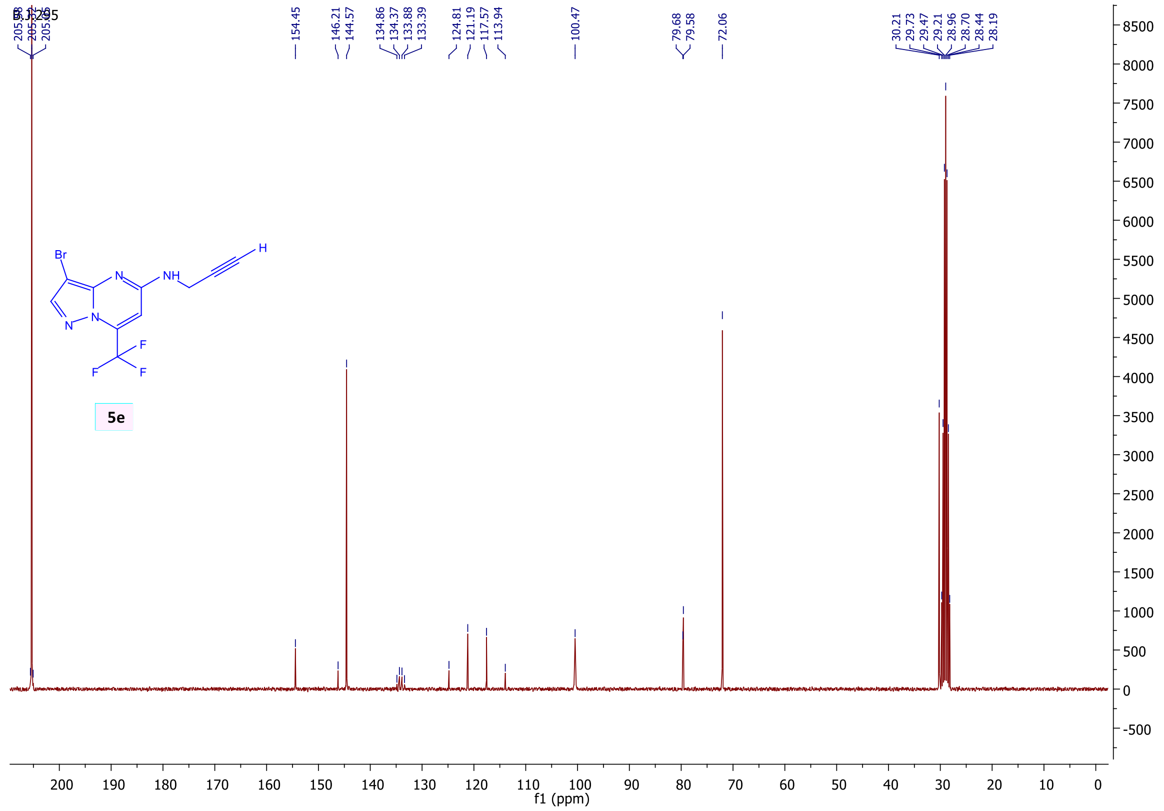

3-Bromo-5-morpholino-7-(trifluoromethyl)pyrazolo[1,5-a]pyrimidine (**5f**)

^{1}H NMR (300 MHz, CDCl$_3$)

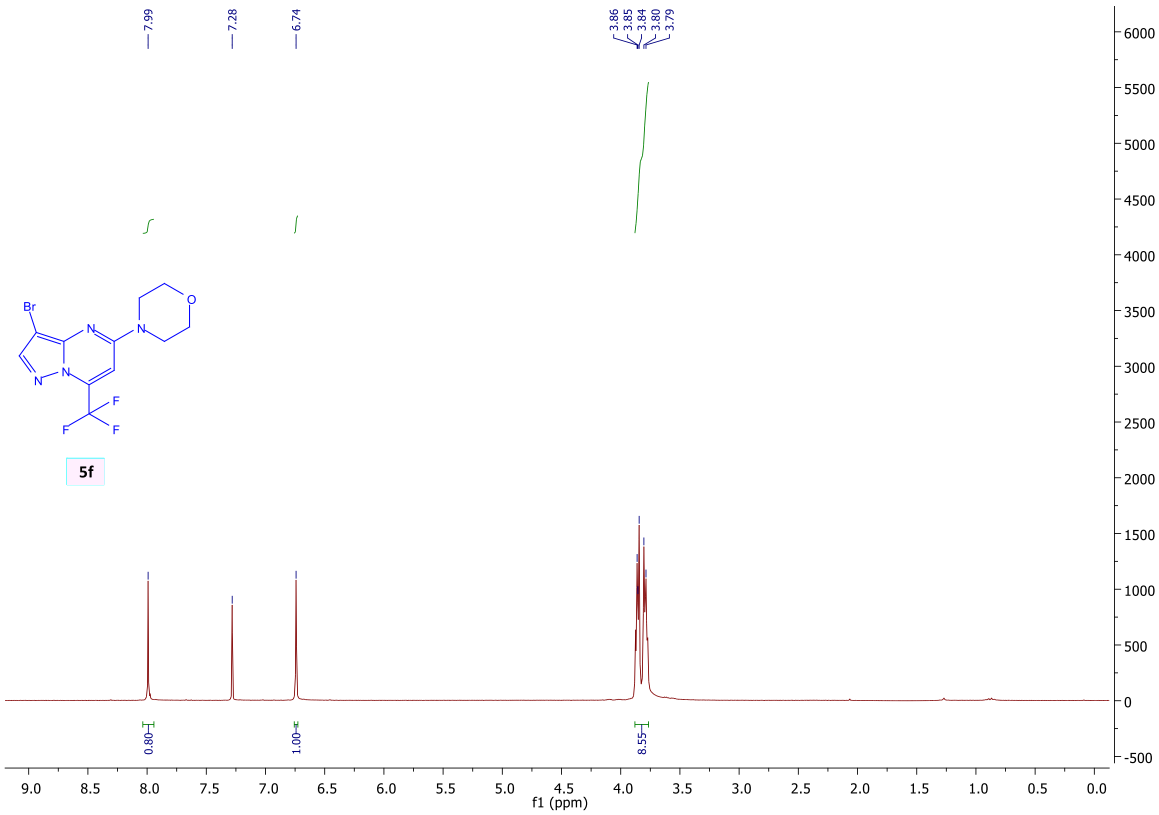

^{19}F NMR (282 MHz, CDCl$_3$)

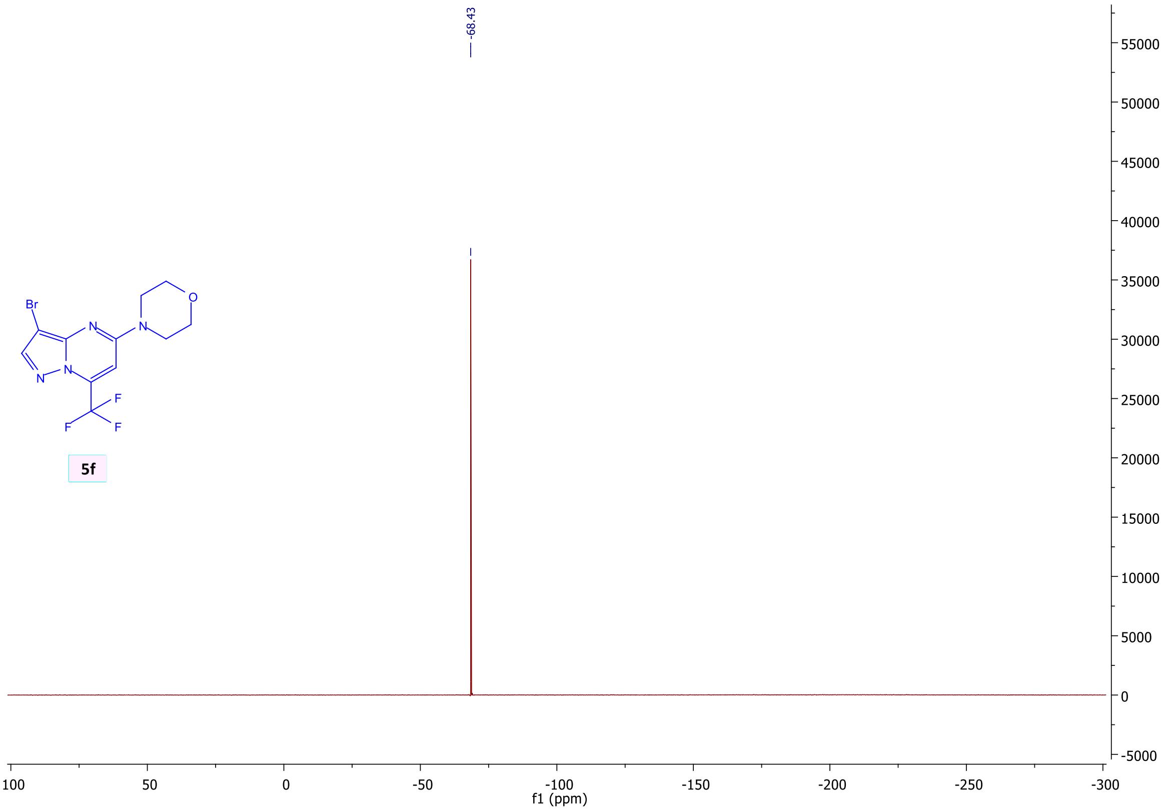

^{13}C NMR (75 MHz, Acétone-*d*$_6$)

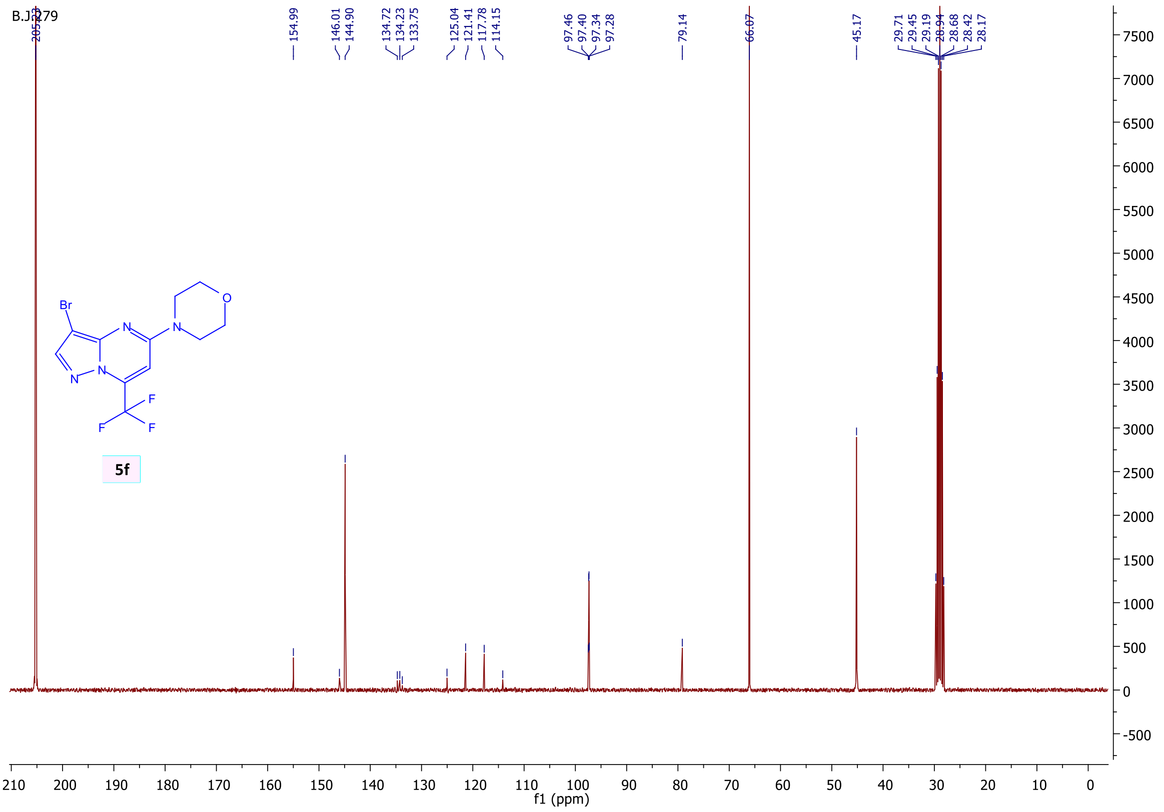

3-bromo-5-((4-methoxyphenyl)thio)-7-(trifluoromethyl)pyrazolo[1,5-a]pyrimidine (**5g**)

^{1}H NMR (300 MHz, CDCl$_3$)

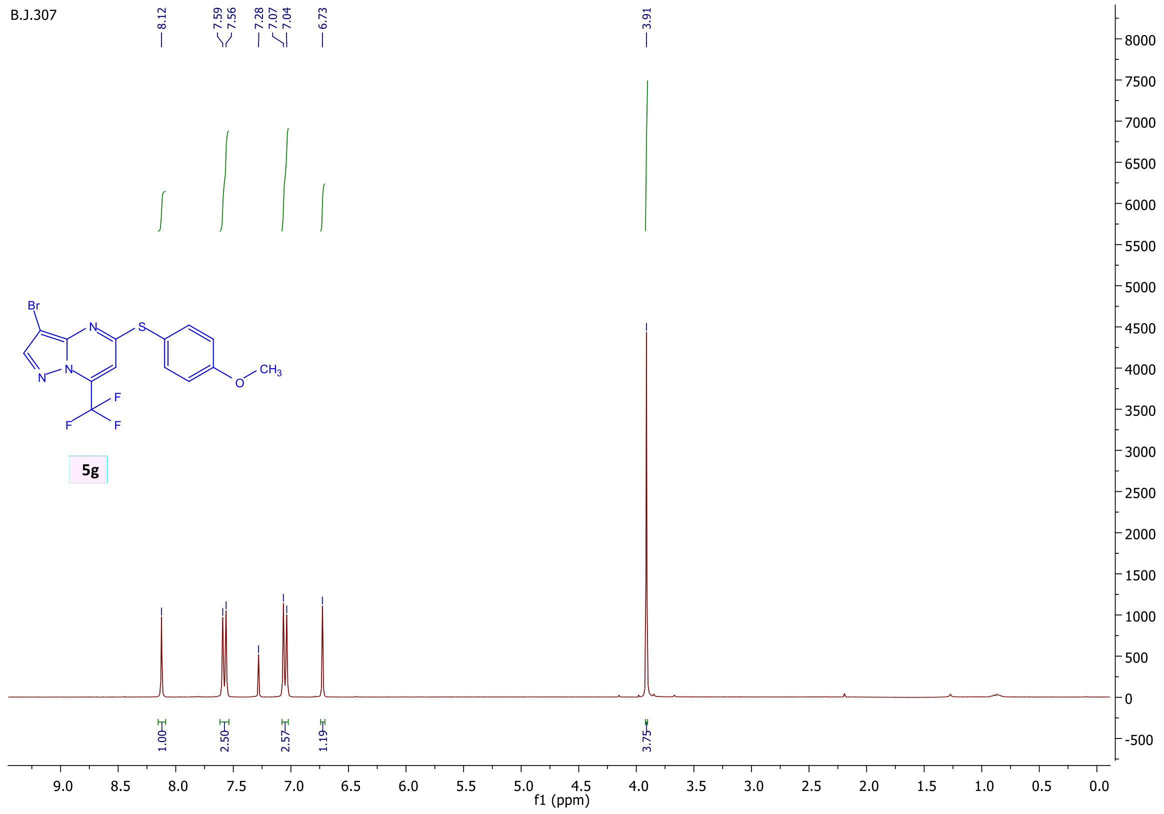

^{19}F NMR (282 MHz, $CDCl_3$)

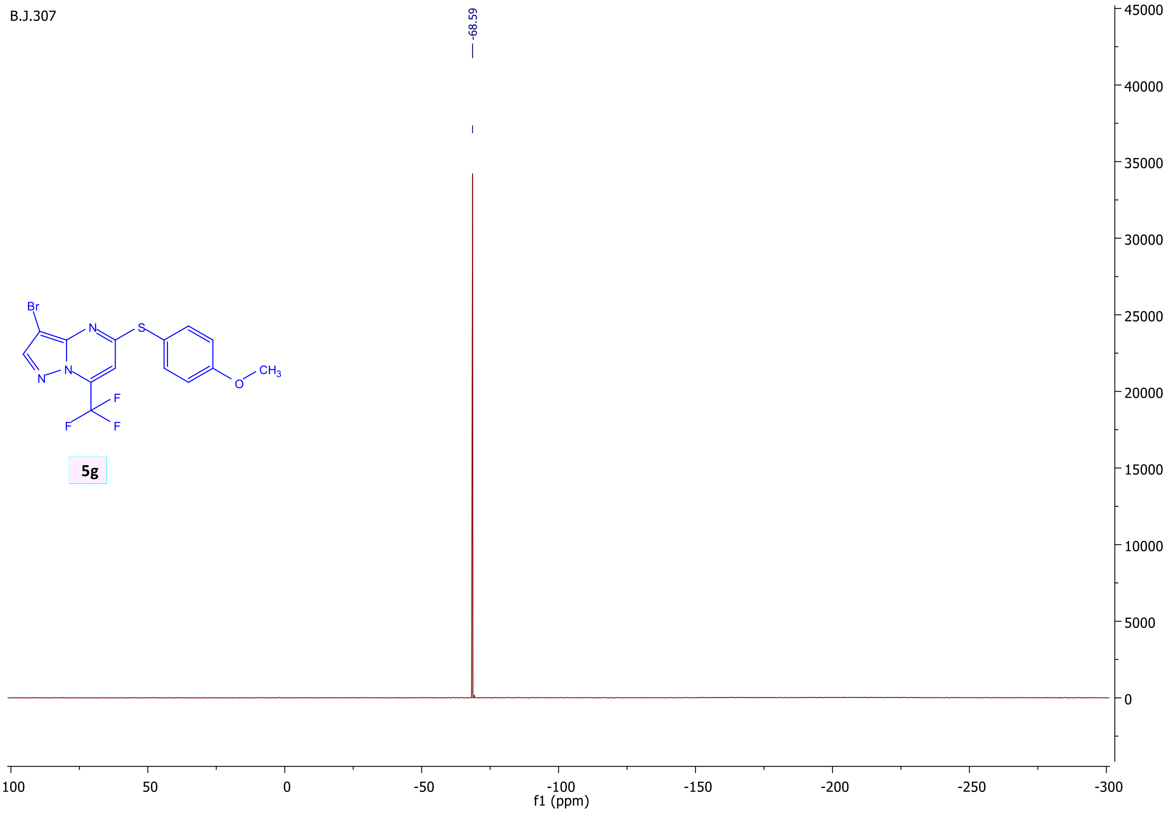

^{13}C NMR (75 MHz, Acétone-*d*$_6$)

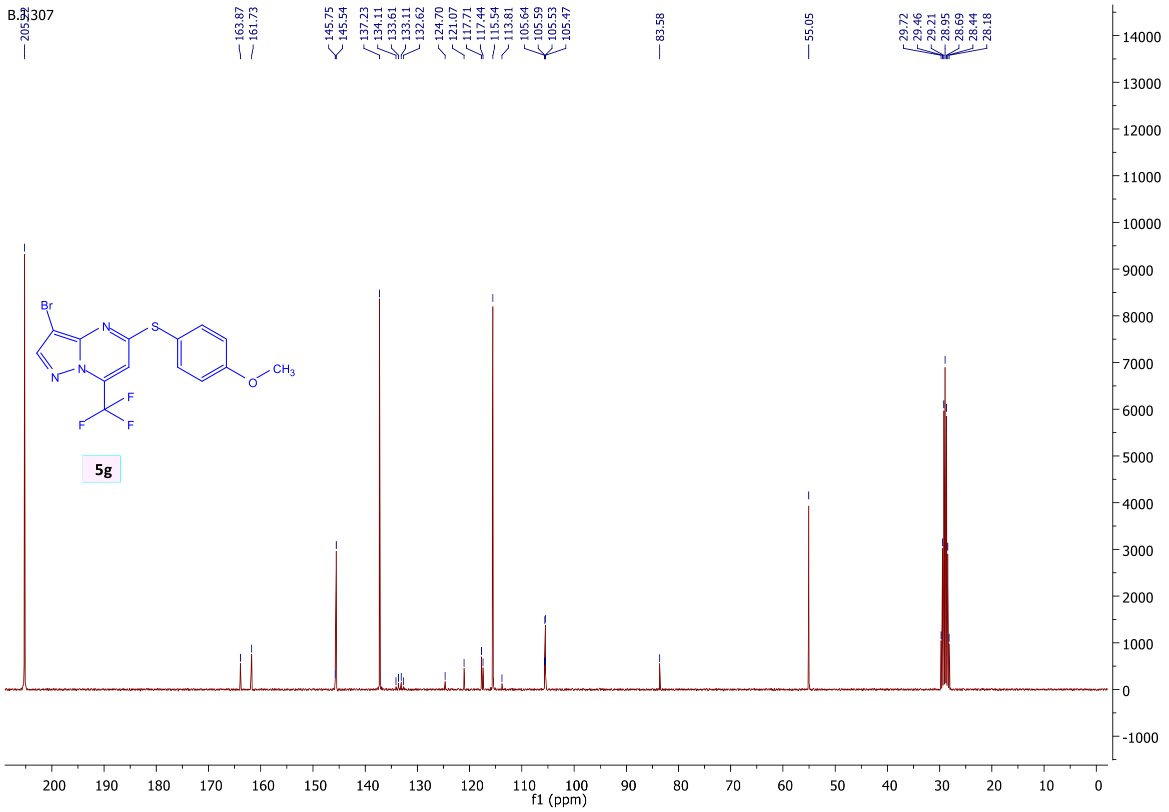

3-(4-methoxyphenyl)-5-morpholine-7-(trifluoromethyl)pyrazolo[1,5-a]pyrimidine (**6a**).

^{1}H NMR (300 MHz, $CDCl_3$)

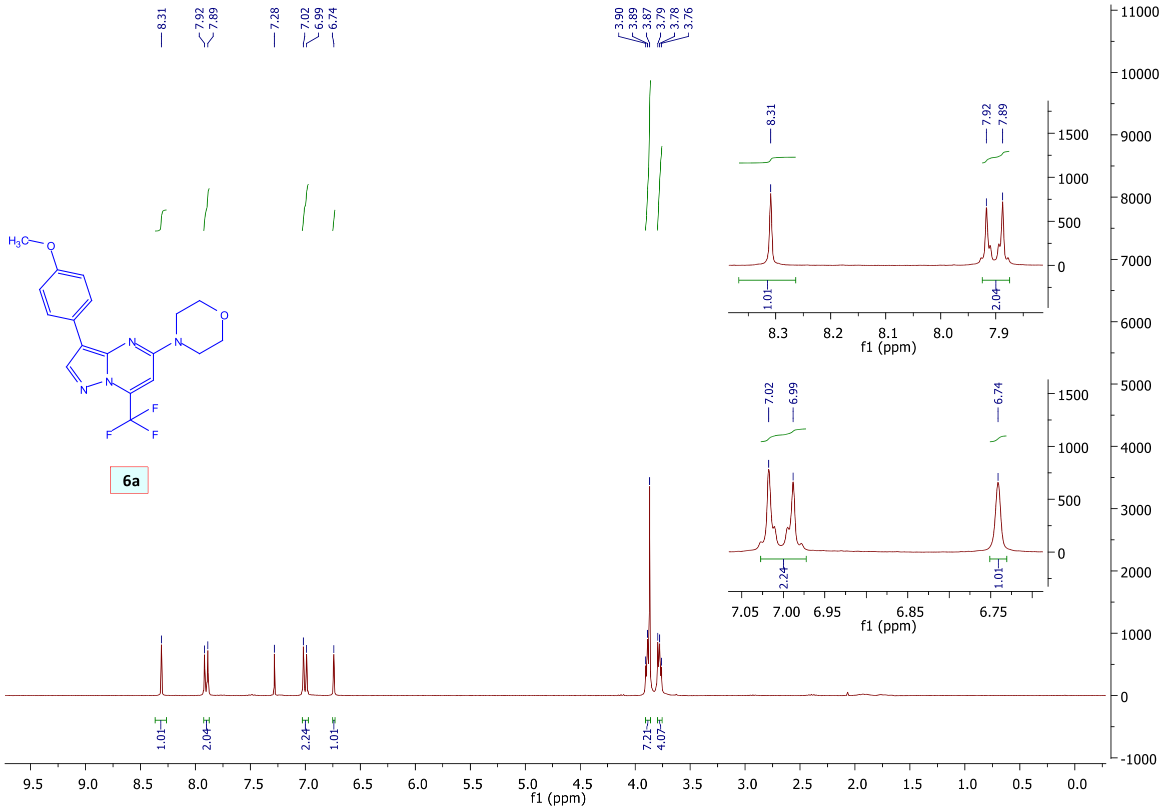

^{19}F NMR (282 MHz, $CDCl_3$)

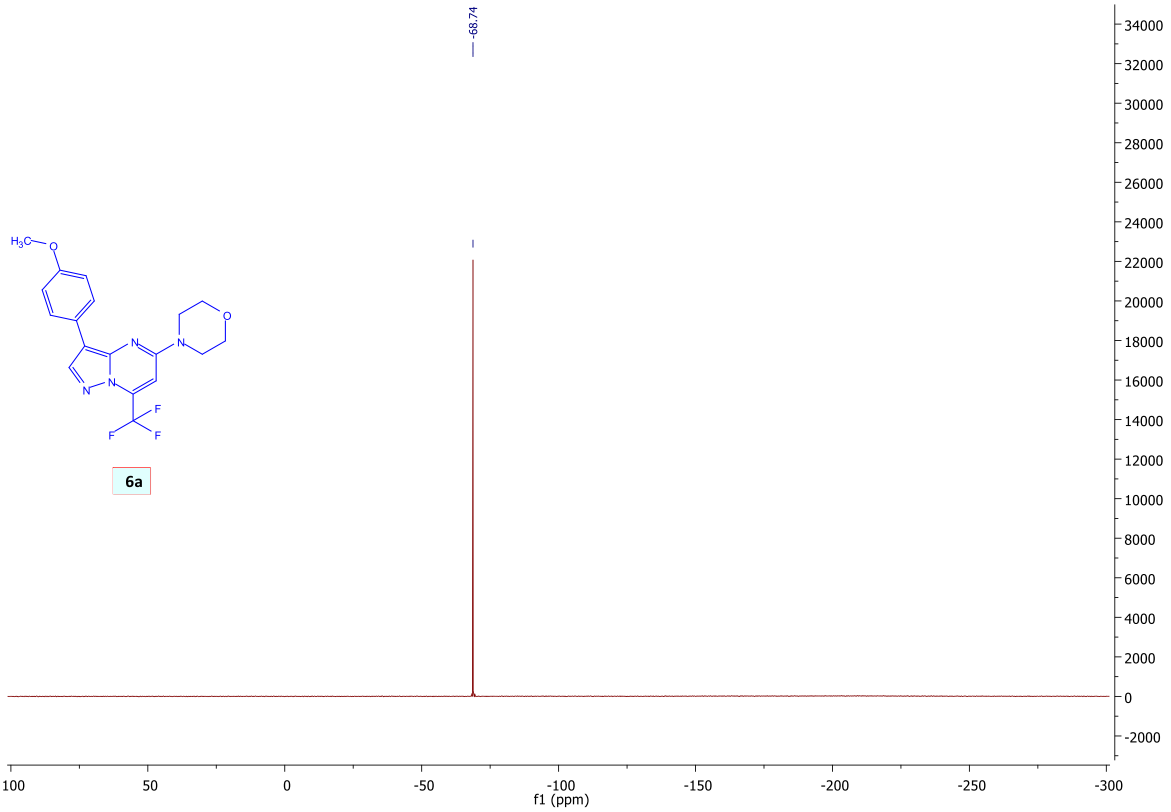

^{13}C NMR (75 MHz, $CDCl_3$)

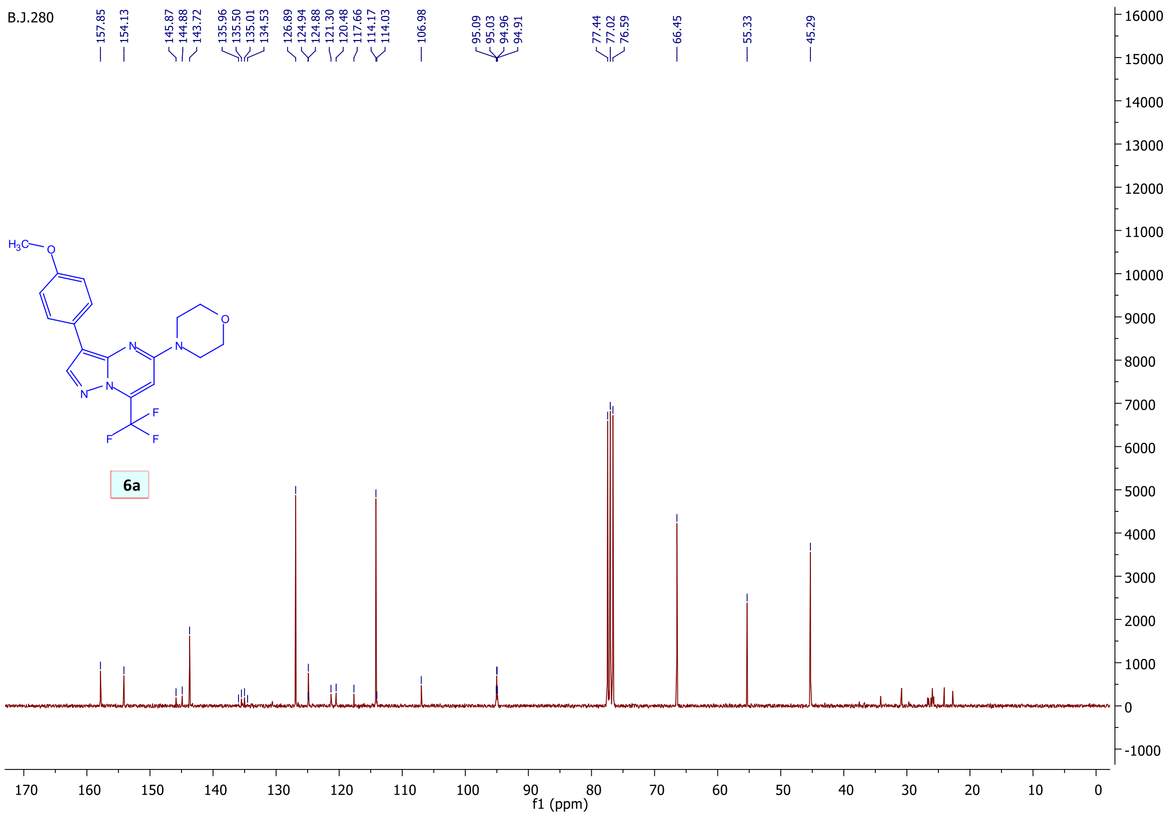

5-Morpholino-3-phenyl-7-(trifluoromethyl)pyrazolo[1,5-a]pyrimidine (**6b**).

^{1}H NMR (300 MHz, $CDCl_3$)

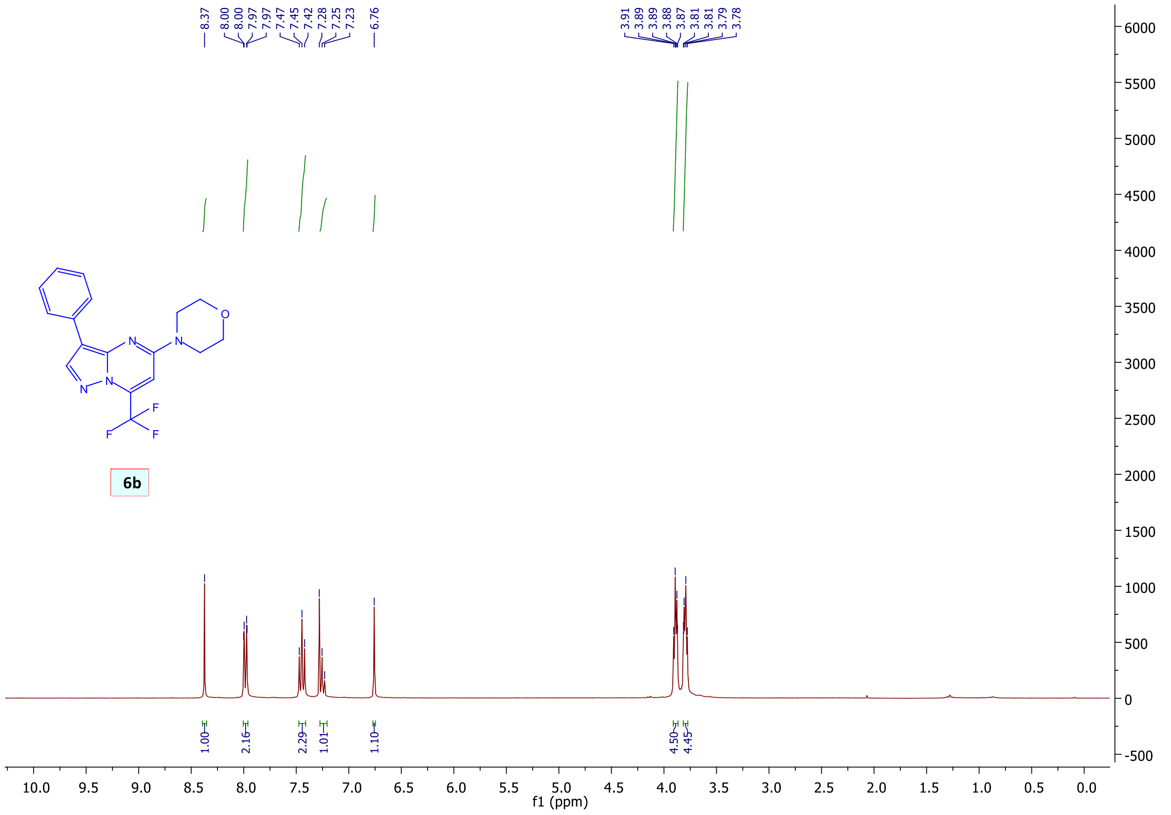

^{19}F NMR (282 MHz, $CDCl_3$)

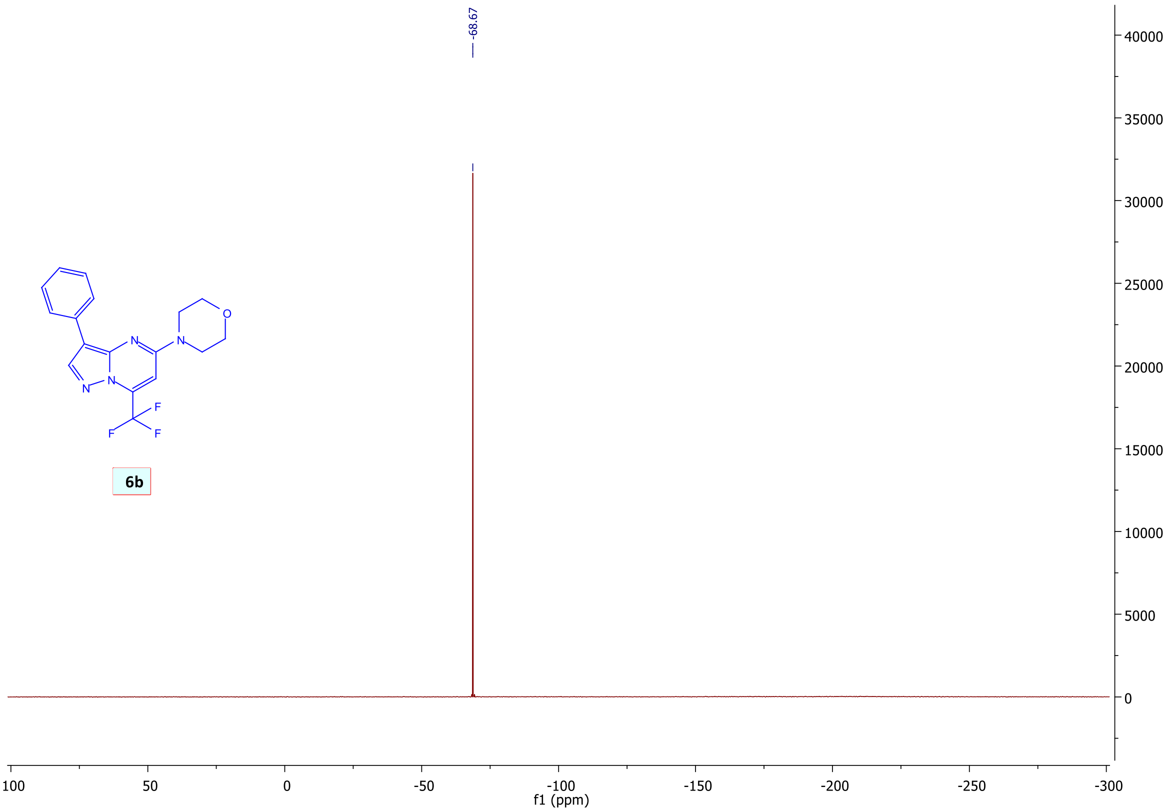

^{13}C NMR (75 MHz, $CDCl_3$)

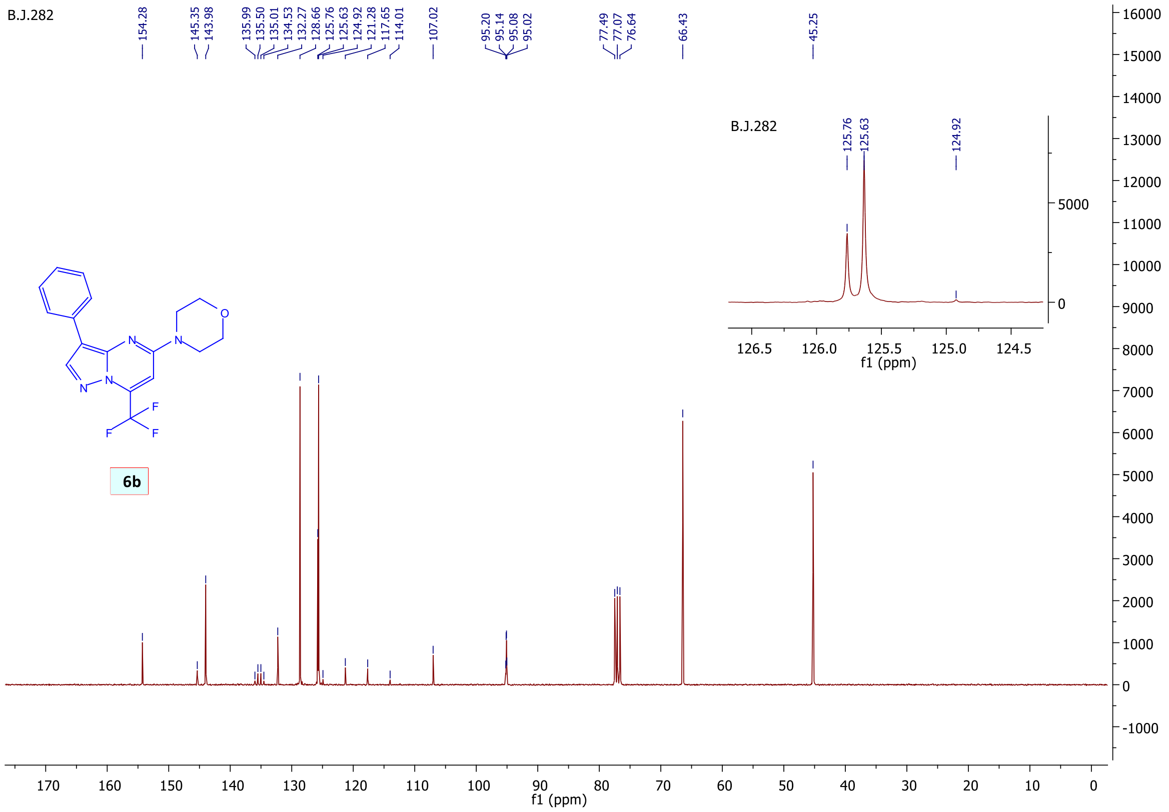

5-Morpholino-3-(thiophen-2-yl)-7-(trifluoromethyl)pyrazolo[1,5-a]pyrimidine (**6c**)

^{1}H NMR (300 MHz, $CDCl_3$)

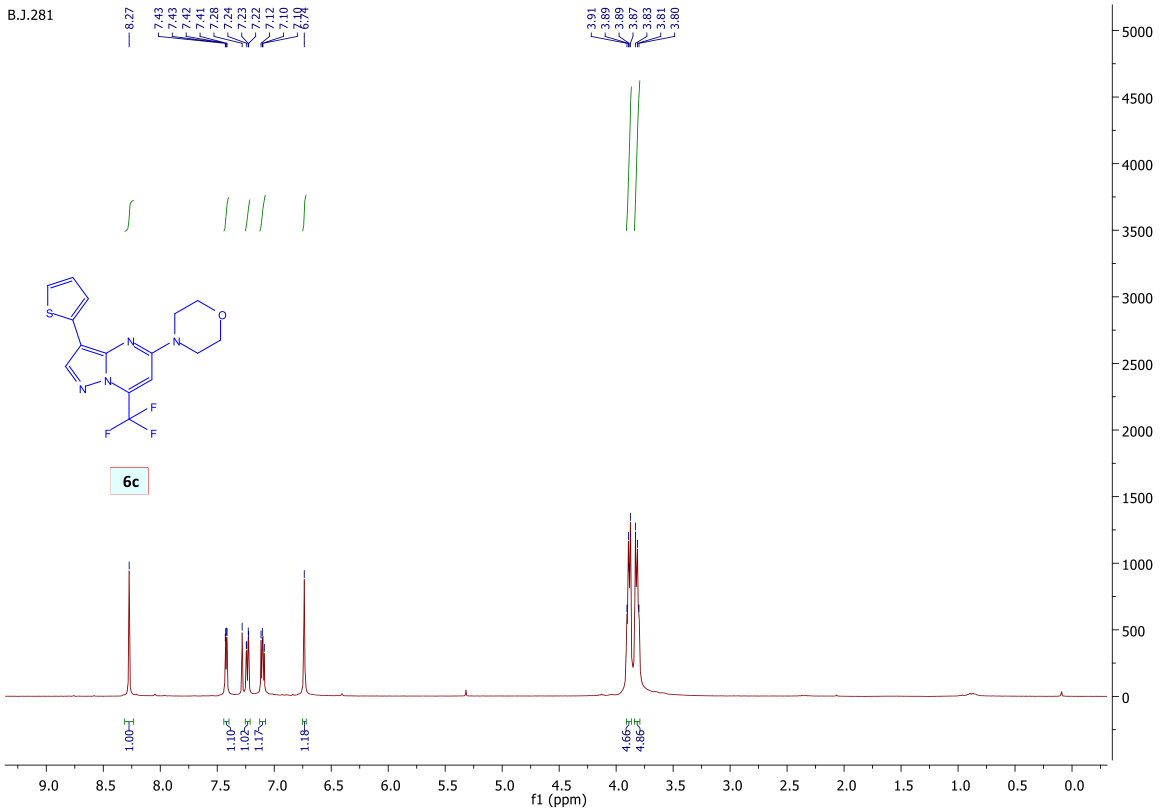

^{19}F NMR (282 MHz, $CDCl_3$)

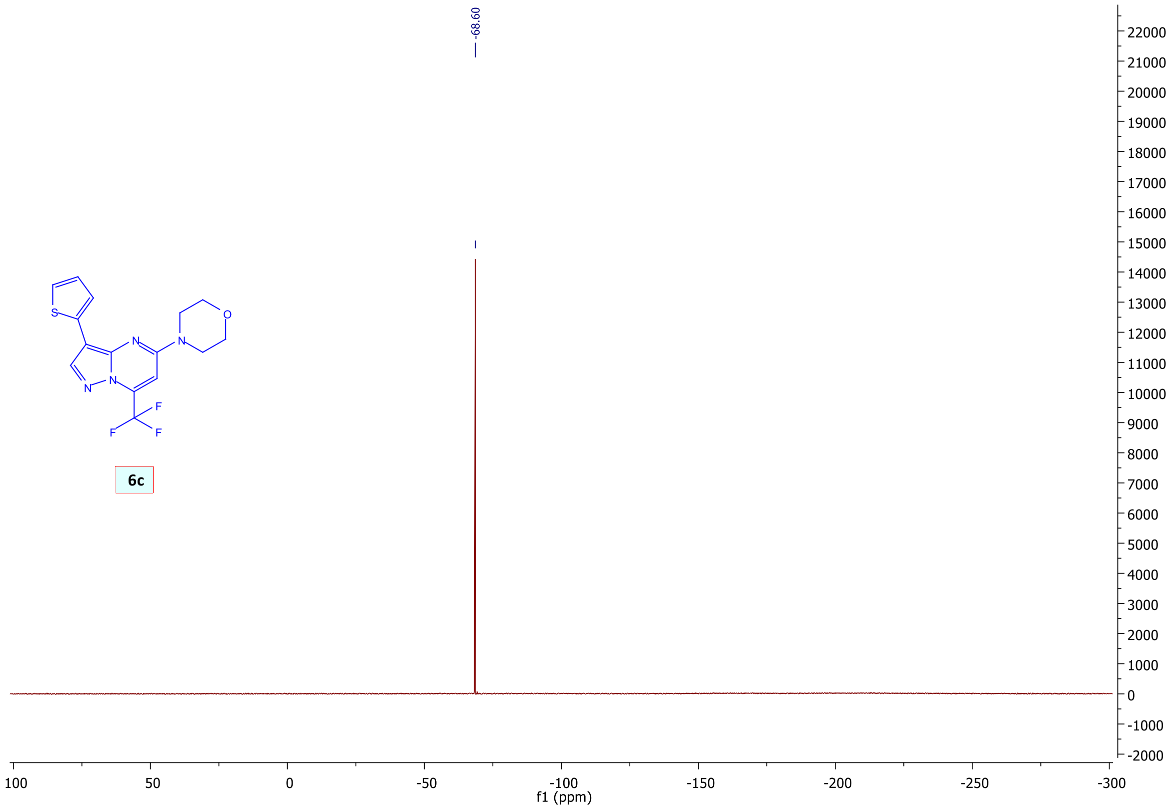

^{13}C NMR (75 MHz, $CDCl_3$)

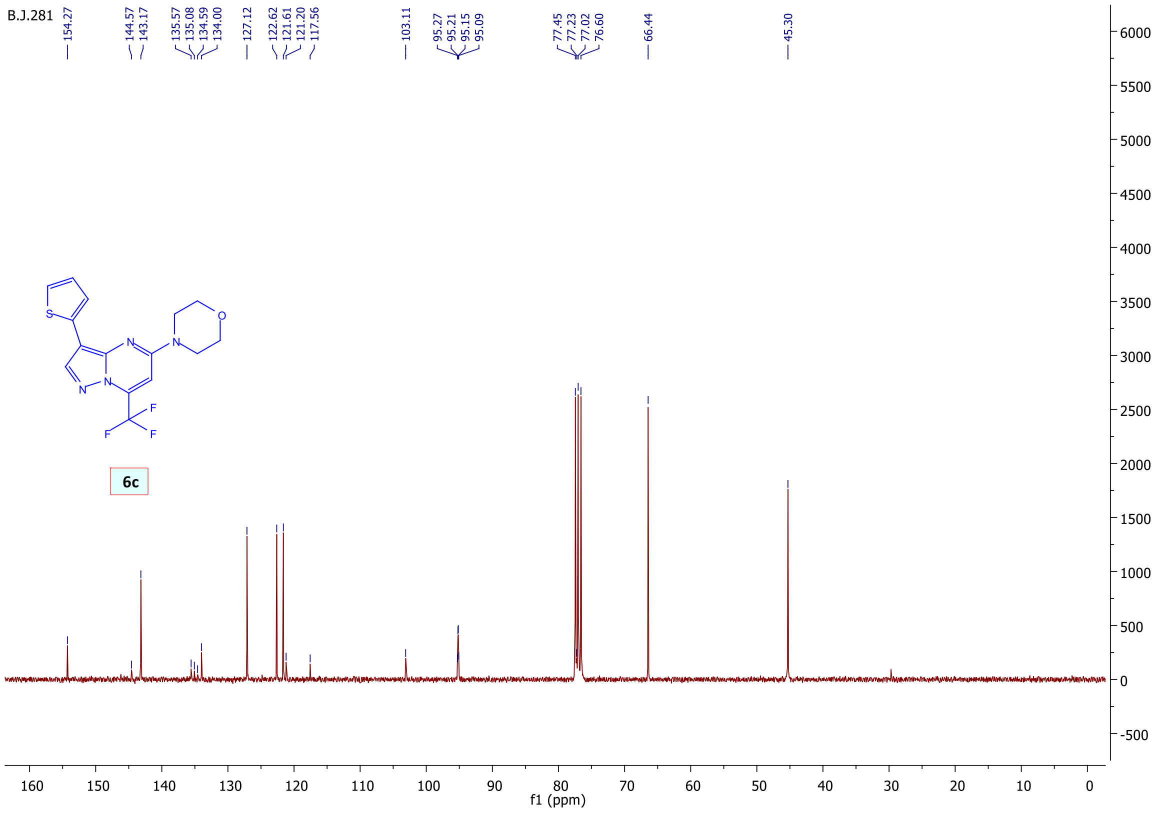

5-(N-Benzylamino)-3-phenyl-7-(trifluoromethyl)pyrazolo[1,5-a]pyrimidine (**6d**)

^{1}H NMR (300 MHz, $CDCl_3$)

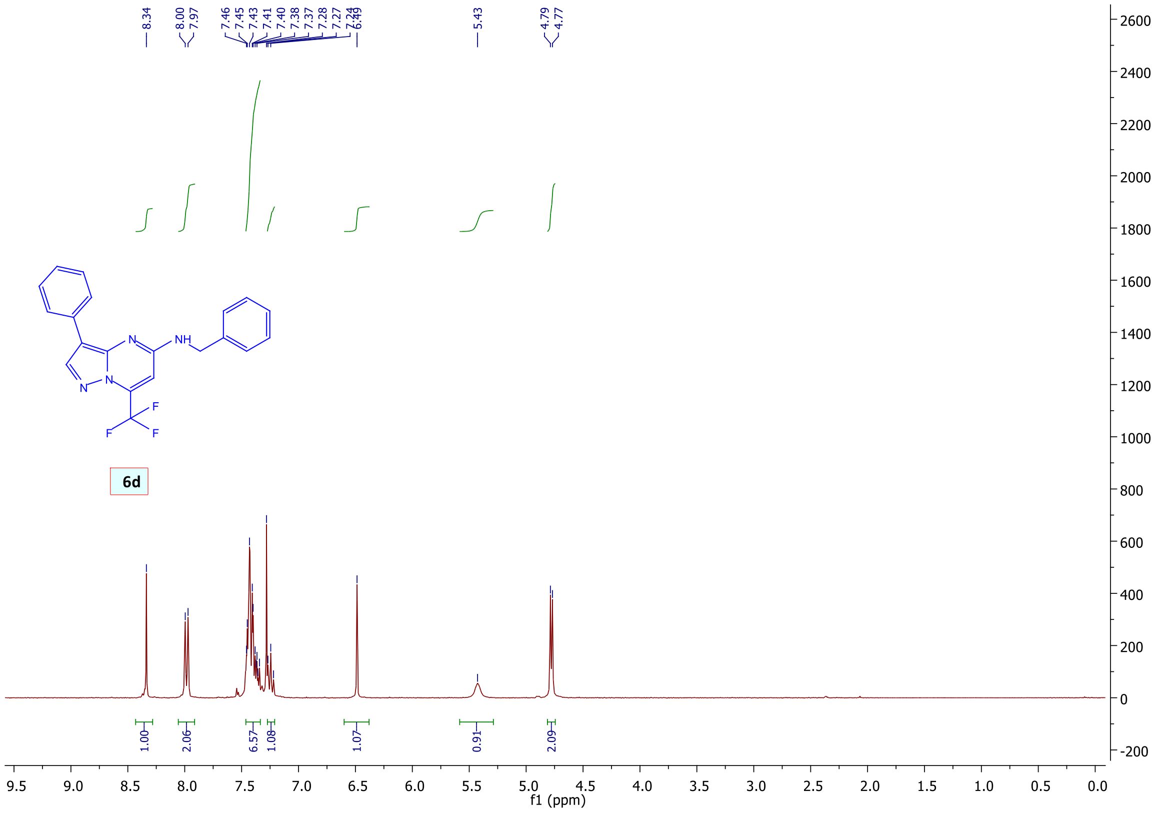

^{19}F NMR (282 MHz, $CDCl_3$)

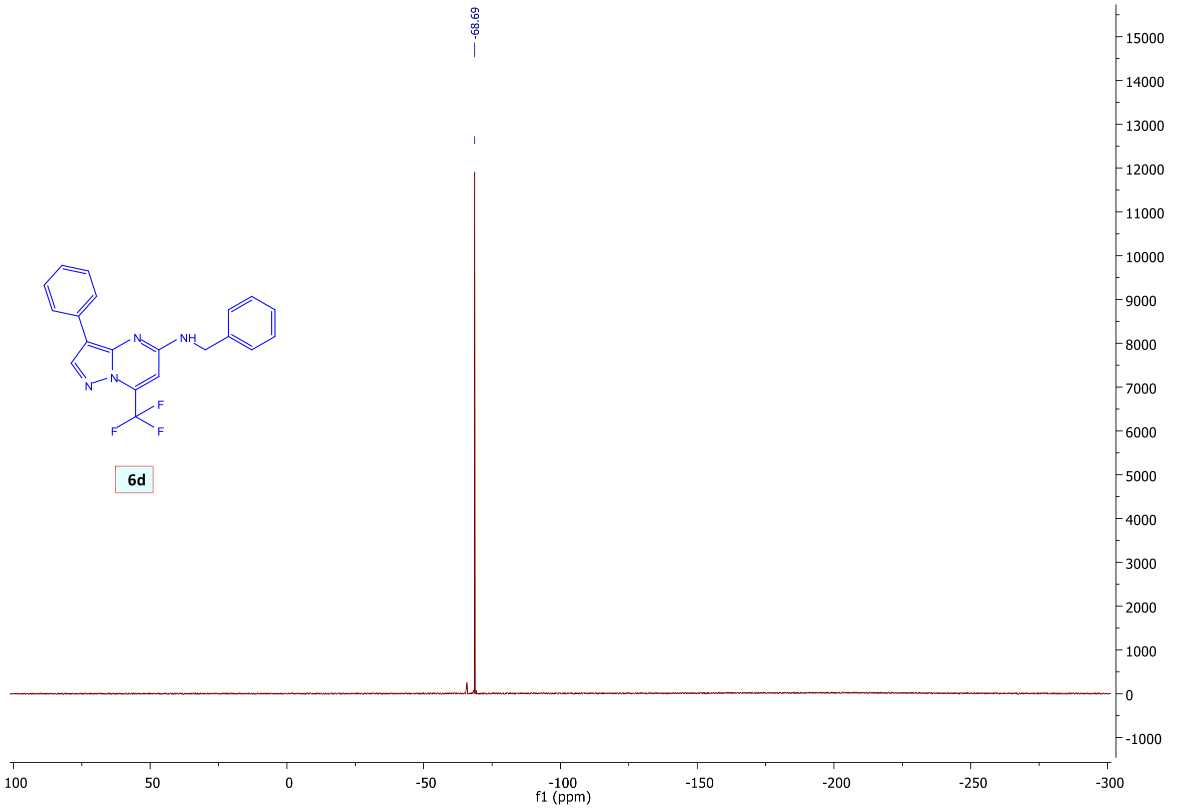

^{13}C NMR (75 MHz, $CDCl_3$)

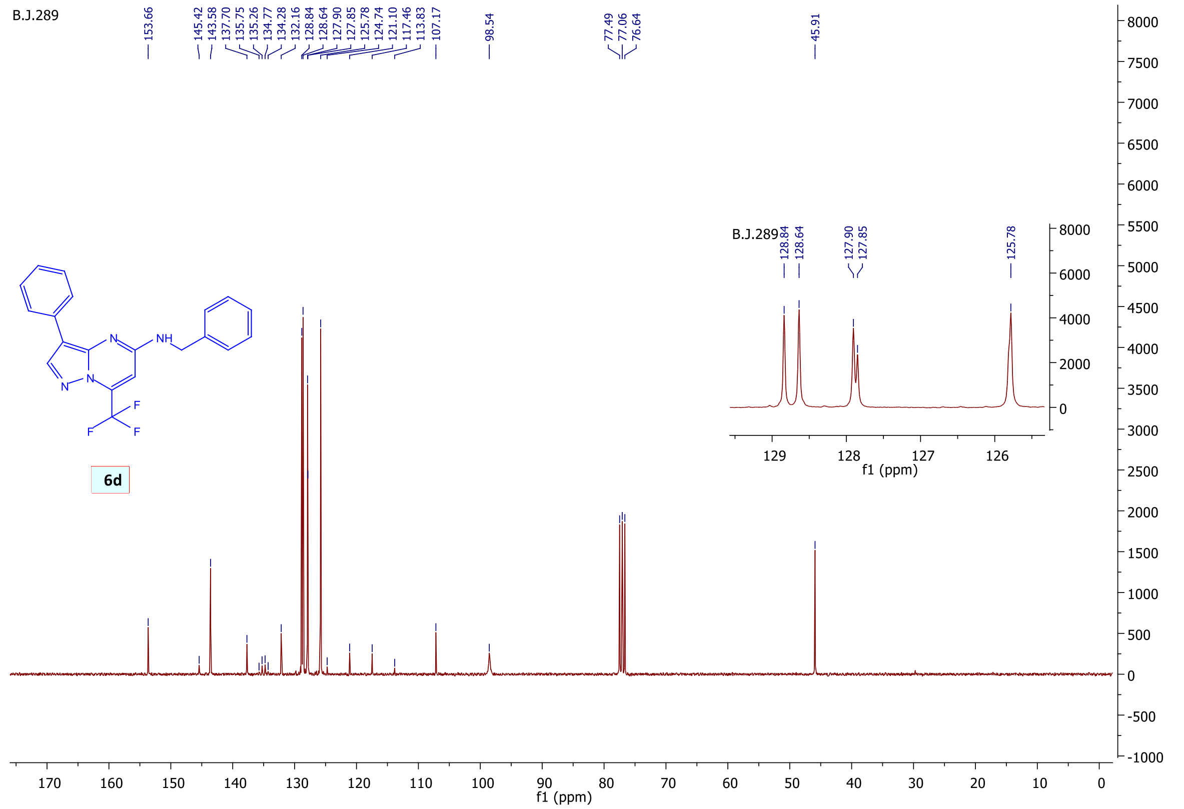

5-(N-Benzylamino)-3-(thiophen-2-yl)-7-(trifluoromethyl)pyrazolo[1,5-a]pyrimidine (**6e**)

^{1}H NMR (300 MHz, $CDCl_3$)

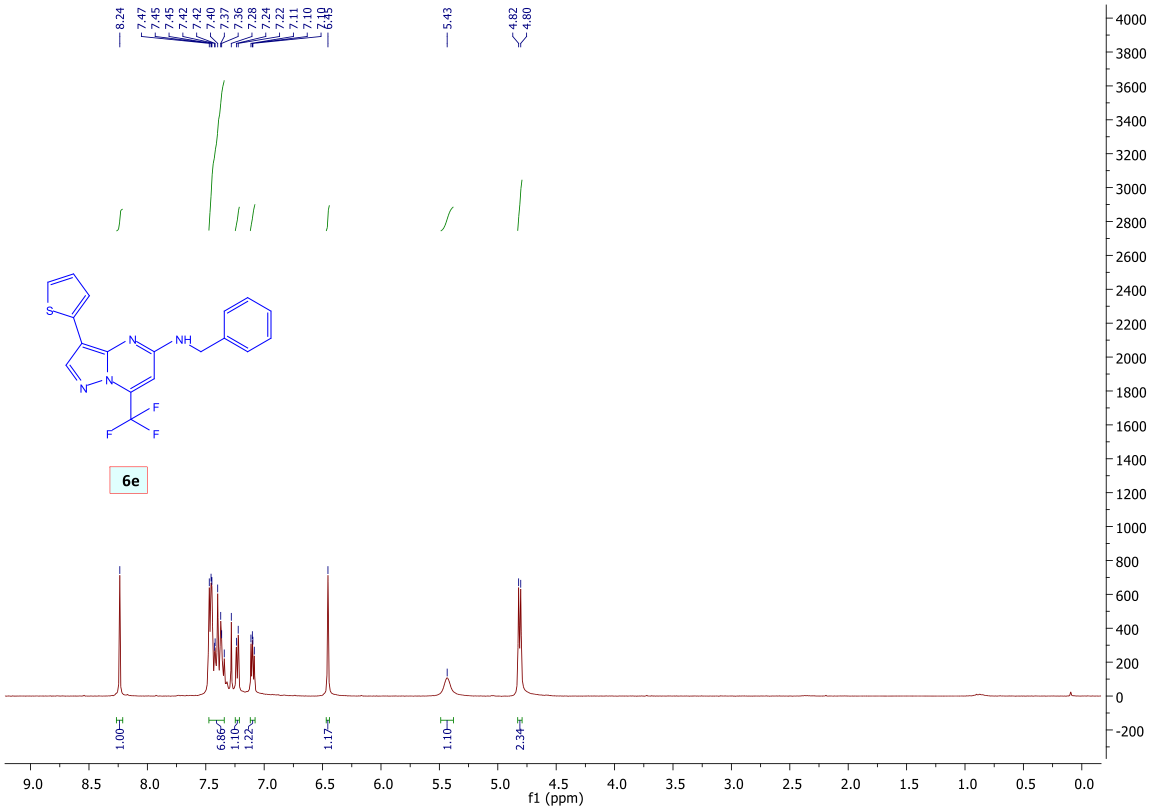

^{19}F NMR (282 MHz, $CDCl_3$)

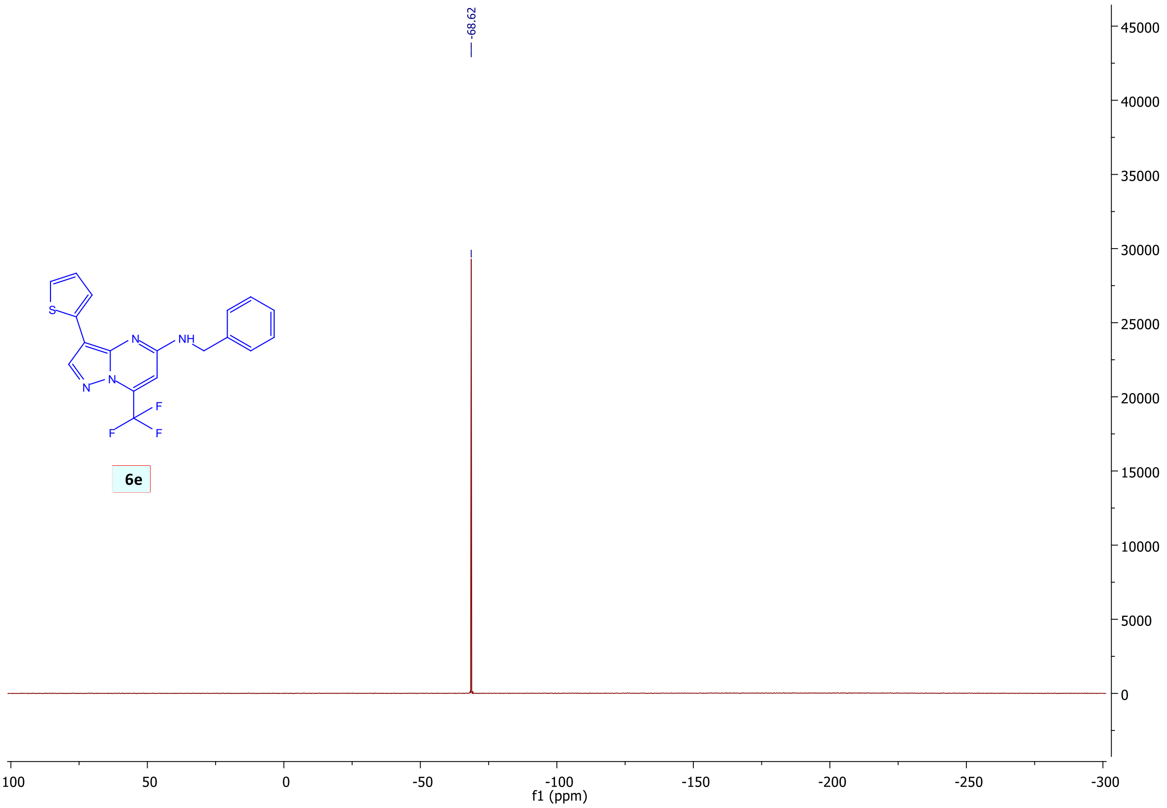

^{13}C NMR (75 MHz, $CDCl_3$)

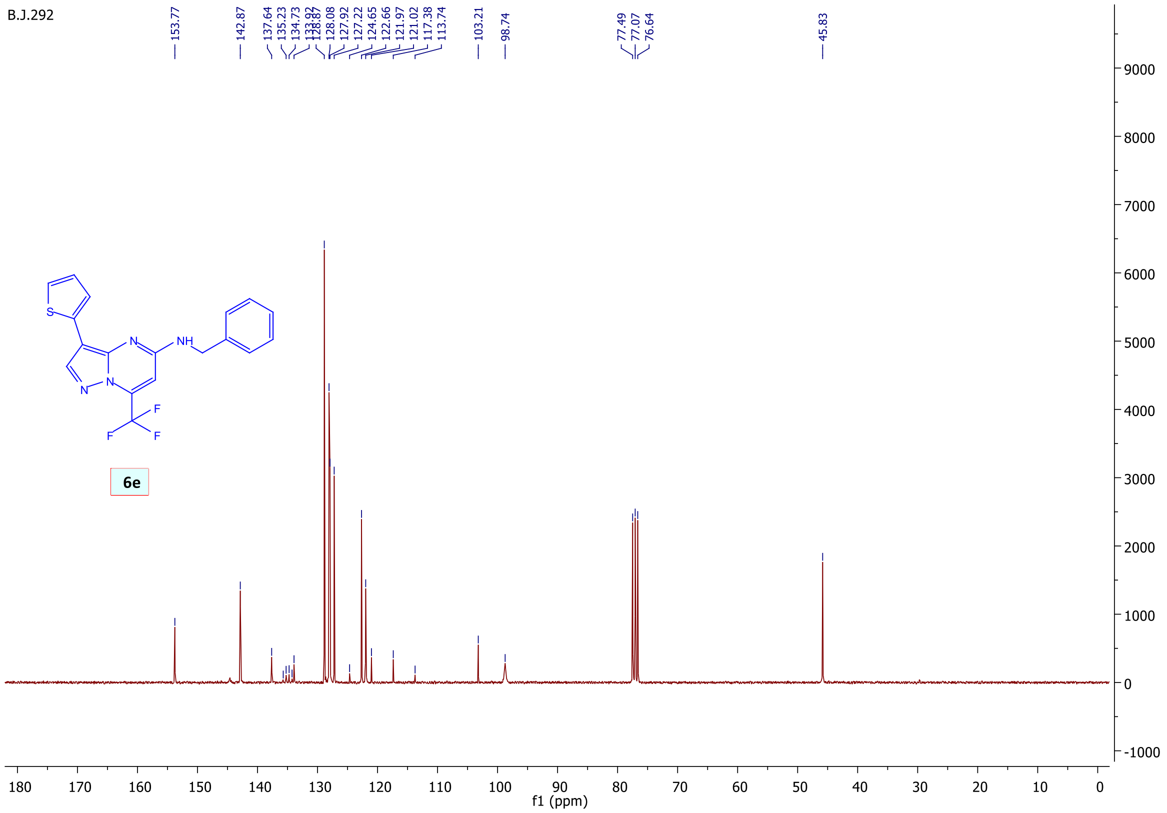

3-([1,1′-Biphenyl]-3-yl)-5-[N-(4-methoxybenzylamino)]-7-(trifluoromethyl)pyrazolo[1,5-a]pyrimidine (**6f**).

^{1}H NMR (300 MHz, $CDCl_3$)

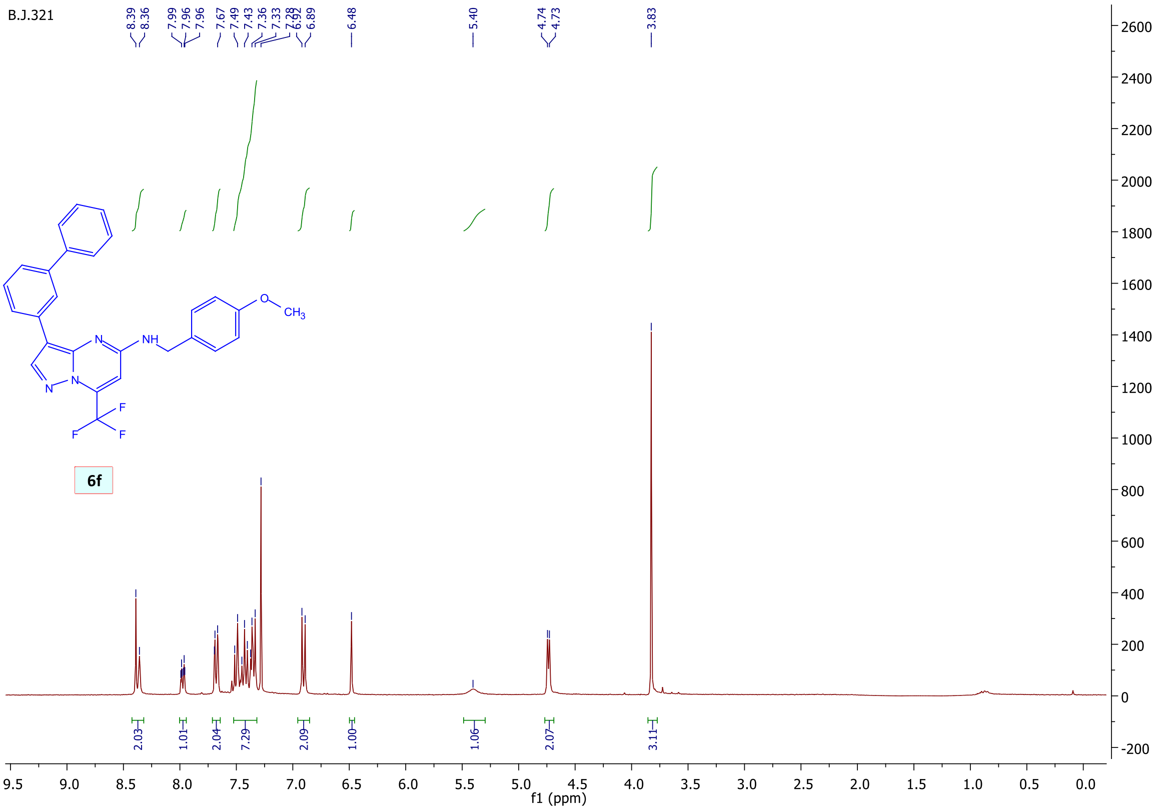

^{19}F NMR (282 MHz, $CDCl_3$)

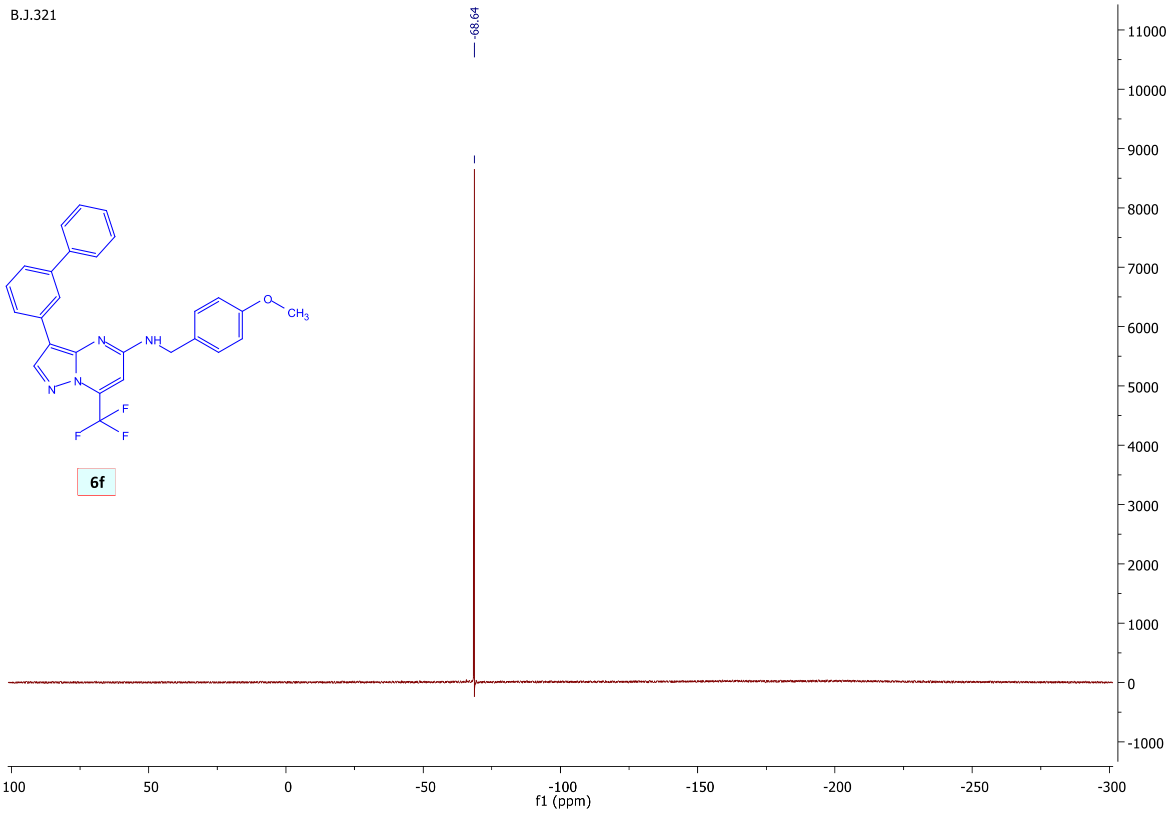

^{13}C NMR (75 MHz, $CDCl_3$)

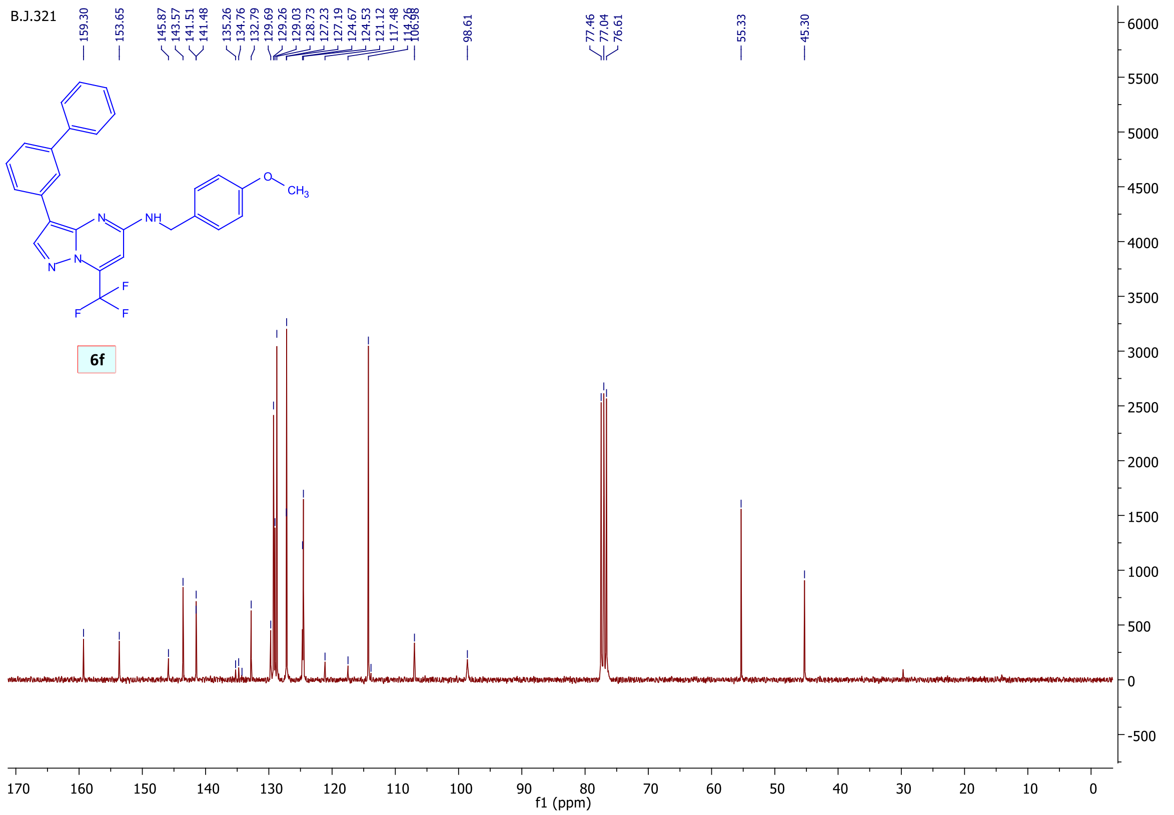

3-[(4-hydroxyméthyl)phenyl]-5-((4-methoxybenzyl)amino)-7-(trifluoromethyl)pyrazolo[1,5-a]pyrimidine (**6g**).

^{1}H NMR (300 MHz, Acétone-*d*$_6$)

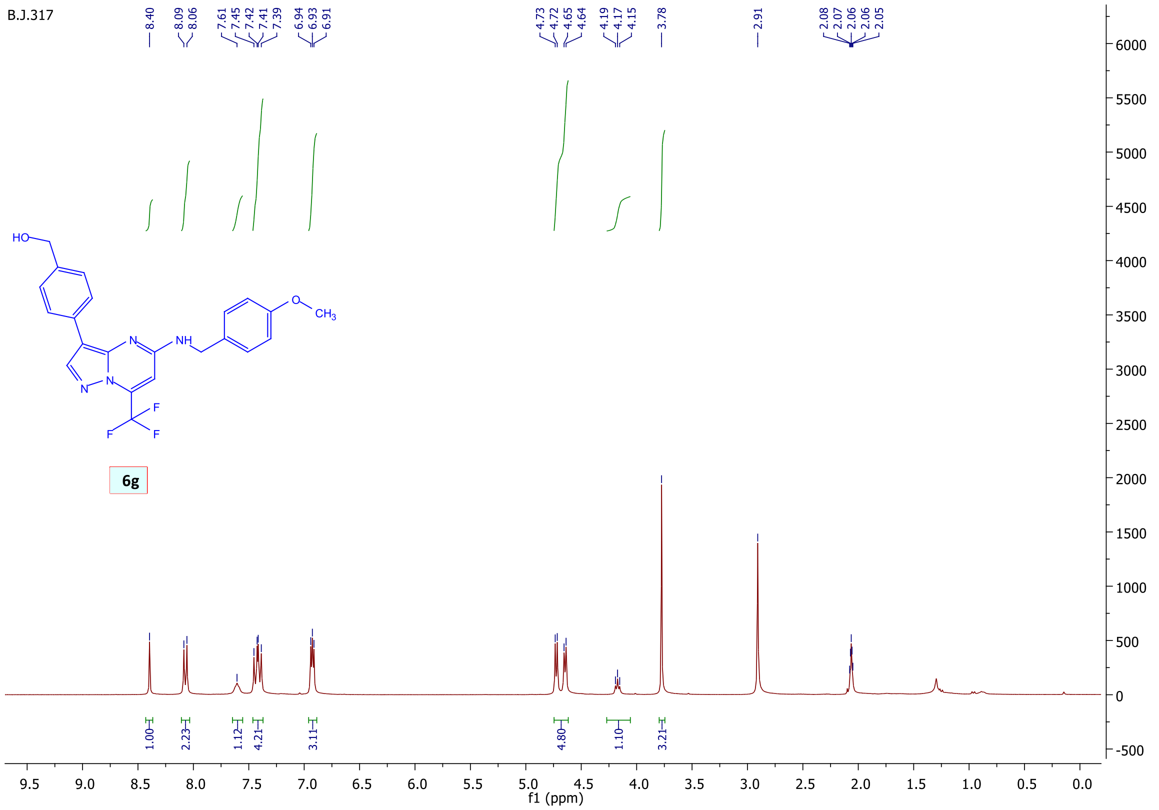

^{19}F NMR (282 MHz, Acétone-*d*$_6$)

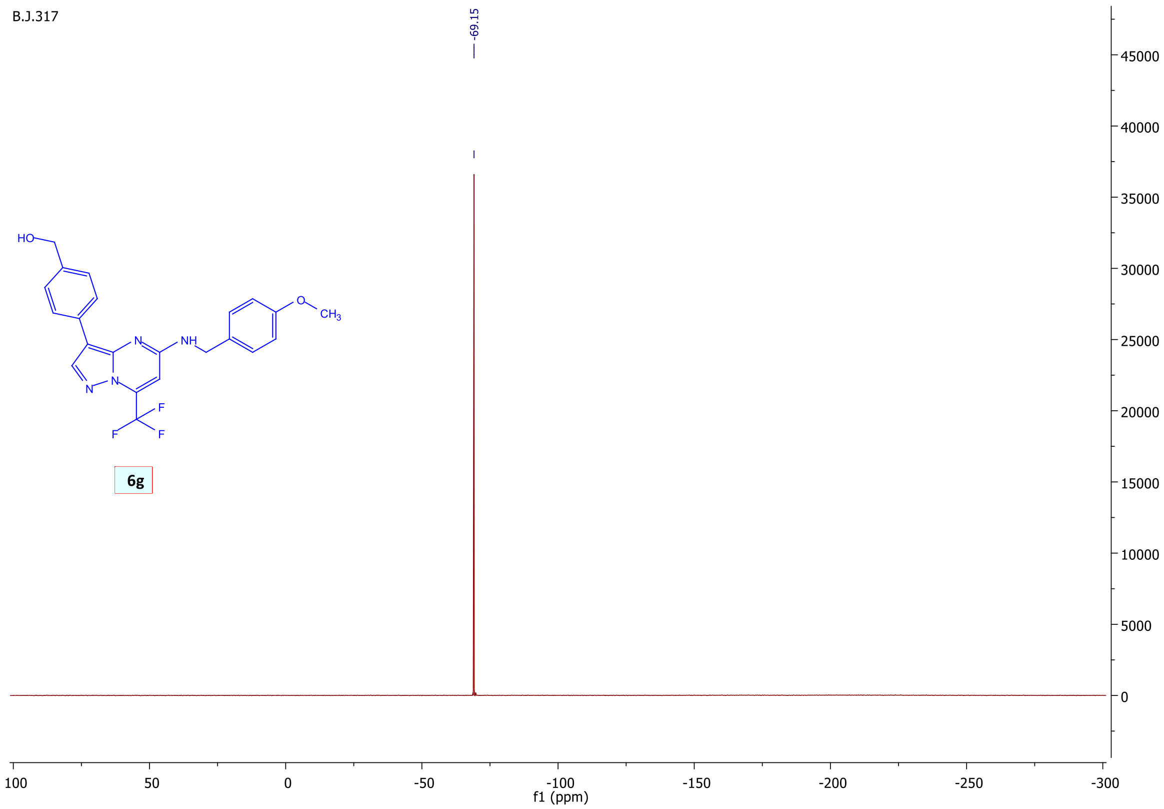

^{13}C NMR (75 MHz, Acétone-*d*$_6$)

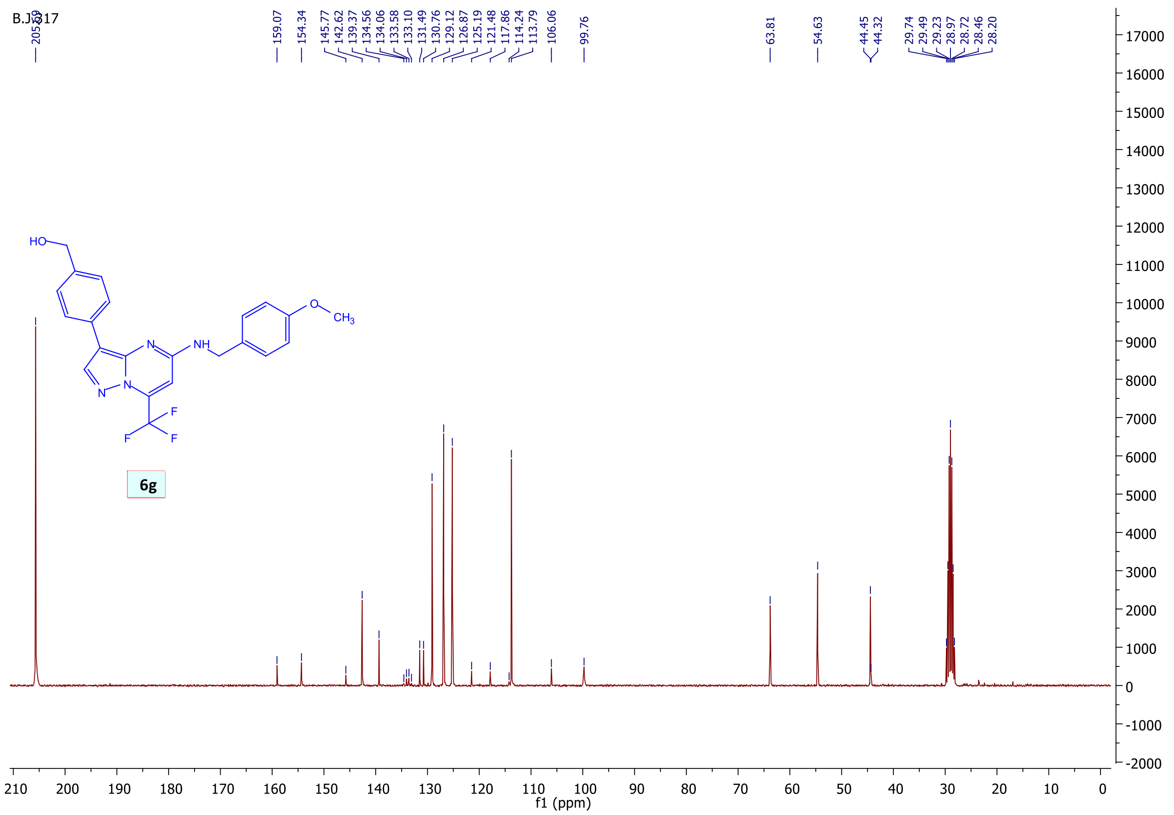

5-[N-(4-methoxybenzyl)amino]-3-(1H-pyrazol-4-yl)-7-(trifluoromethyl)pyrazolo[1,5-a]pyrimidine (**6h**).

^{1}H NMR (300 MHz, Acétone-*d*$_6$)

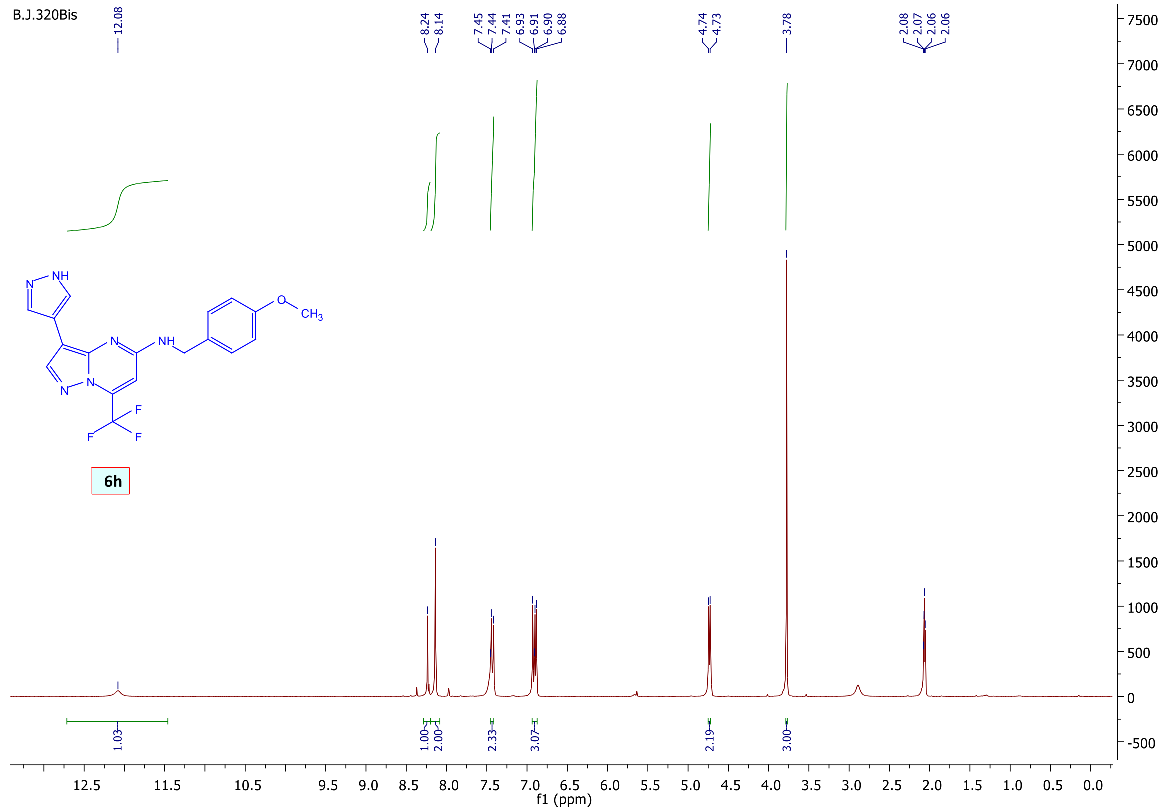

^{19}F NMR (282 MHz, Acétone-d_6).

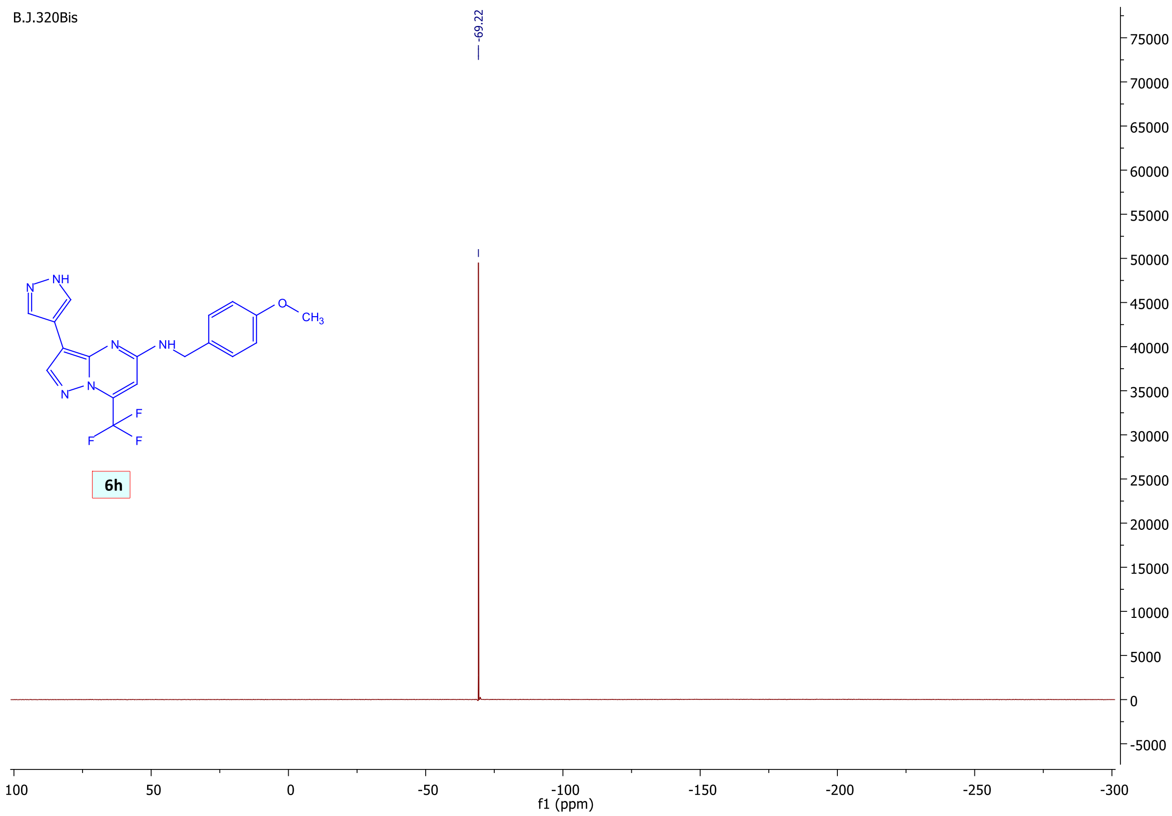

^{13}C NMR (75 MHz, Acétone-d_6)

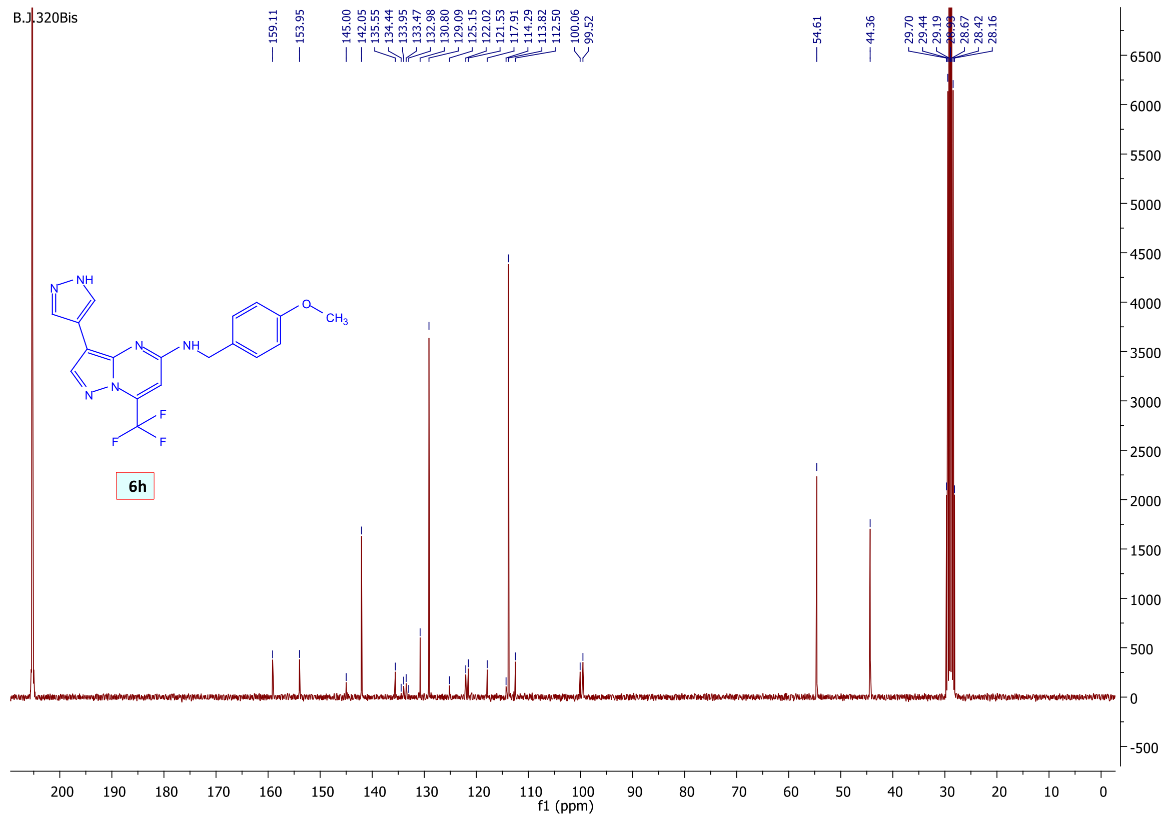

5-[N-(4-Methoxybenzyl)amino]-3-(pyridin-3-yl)-7-(trifluoromethyl)pyrazolo[1,5-a]pyrimidine (**6i**).

^{1}H NMR (300 MHz, $CDCl_3$)

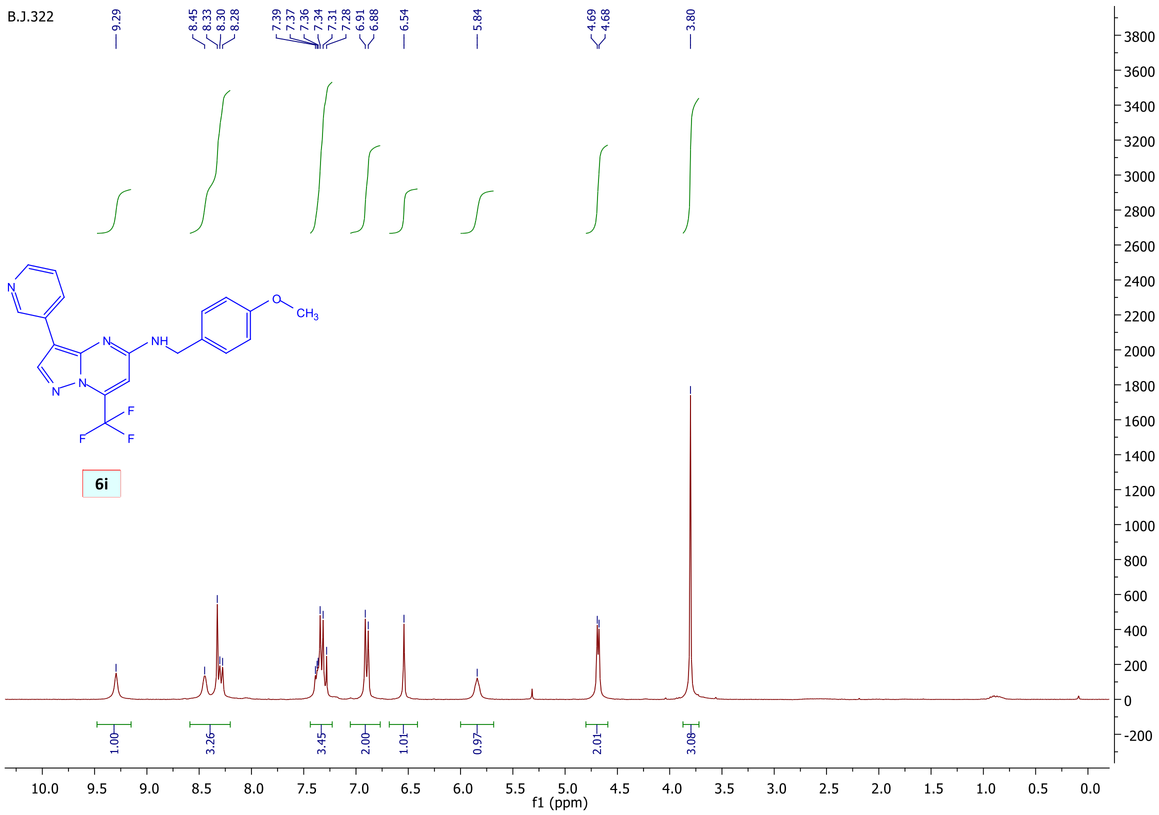

^{19}F NMR (282 MHz, $CDCl_3$)

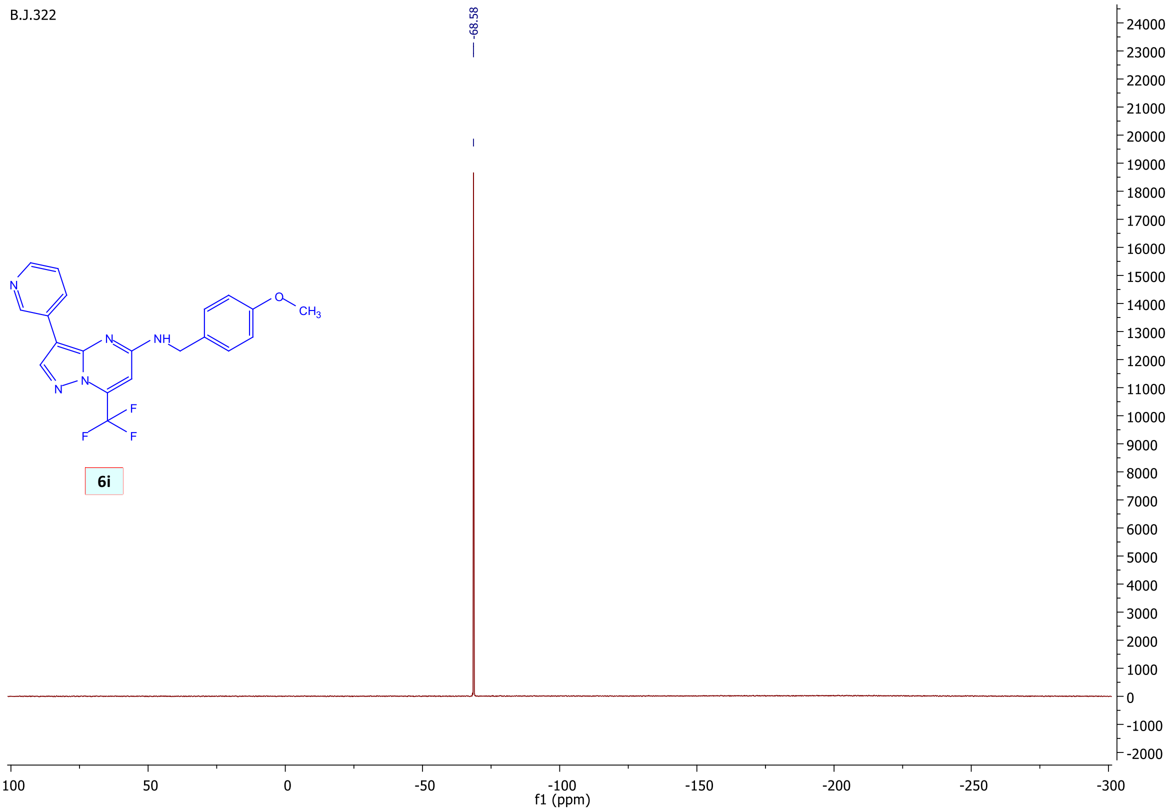

^{13}C NMR (75 MHz, $CDCl_3$)

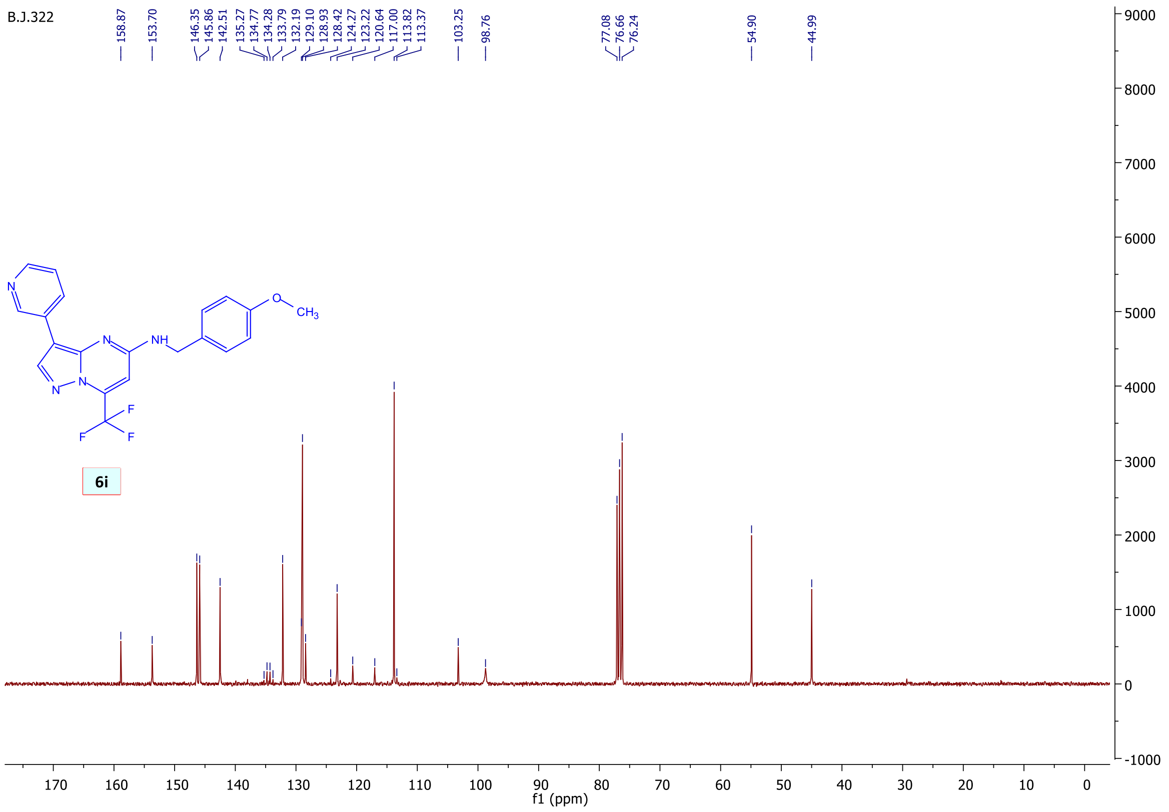

3-(Dibenzo[b,d]thiophen-4-yl)-5-[N-(4-methoxybenzyl)amino]-7-(trifluoromethyl)pyrazolo[1,5-a]pyrimidine (**6j**).

^{1}H NMR (300 MHz, Acétone-d_6)

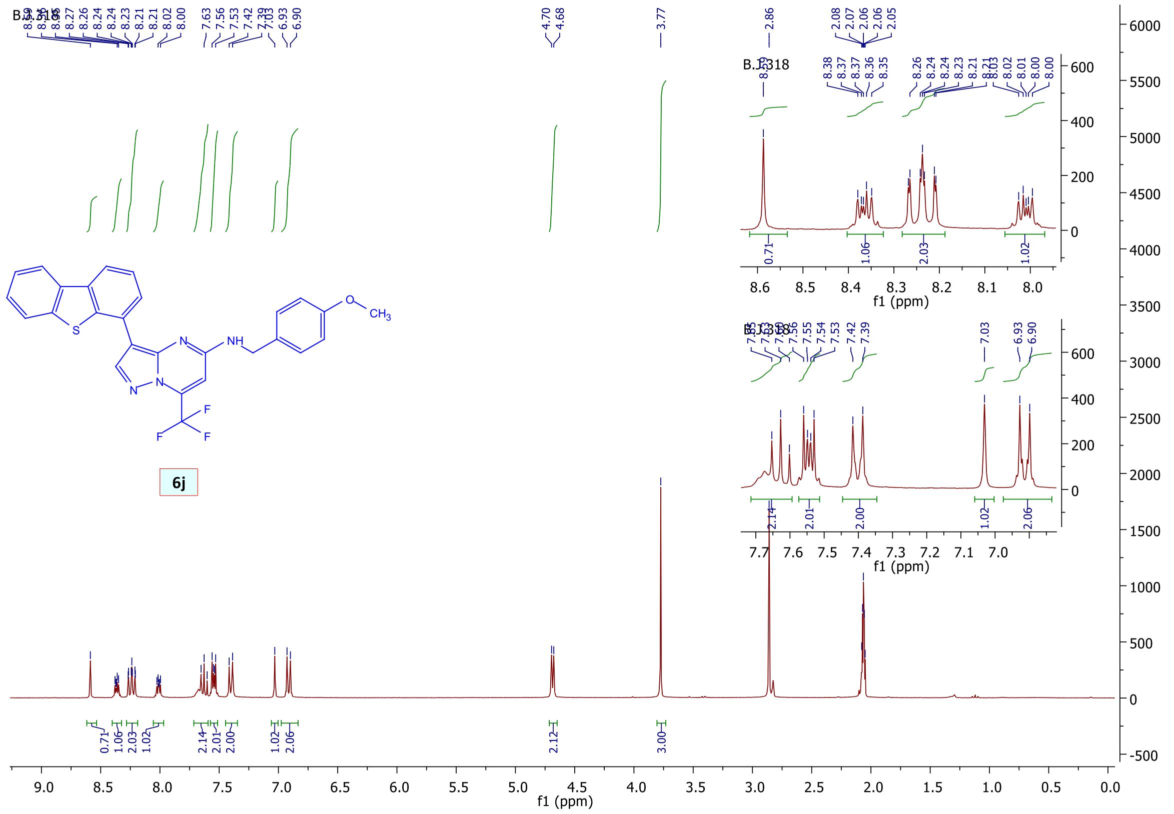

^{19}F NMR (282 MHz, Acétone-d_6)

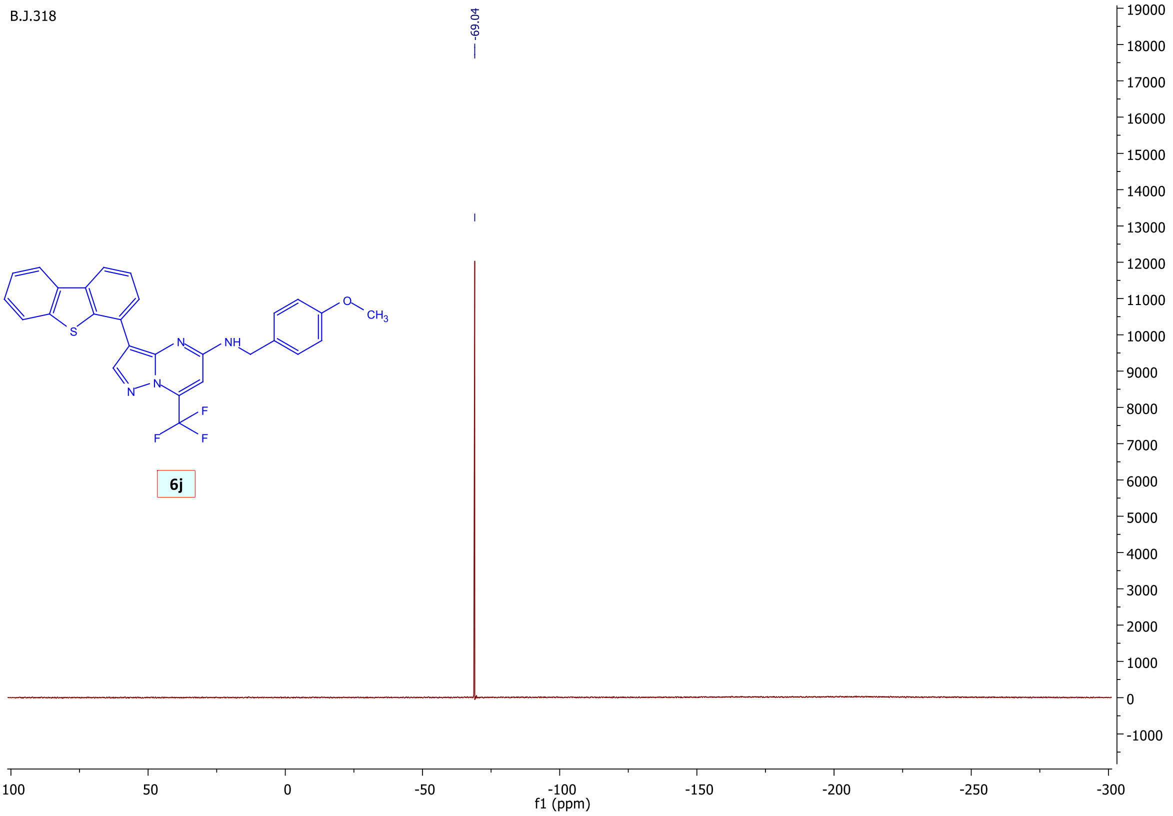

^{13}C NMR (75 MHz, Acétone-d_6)

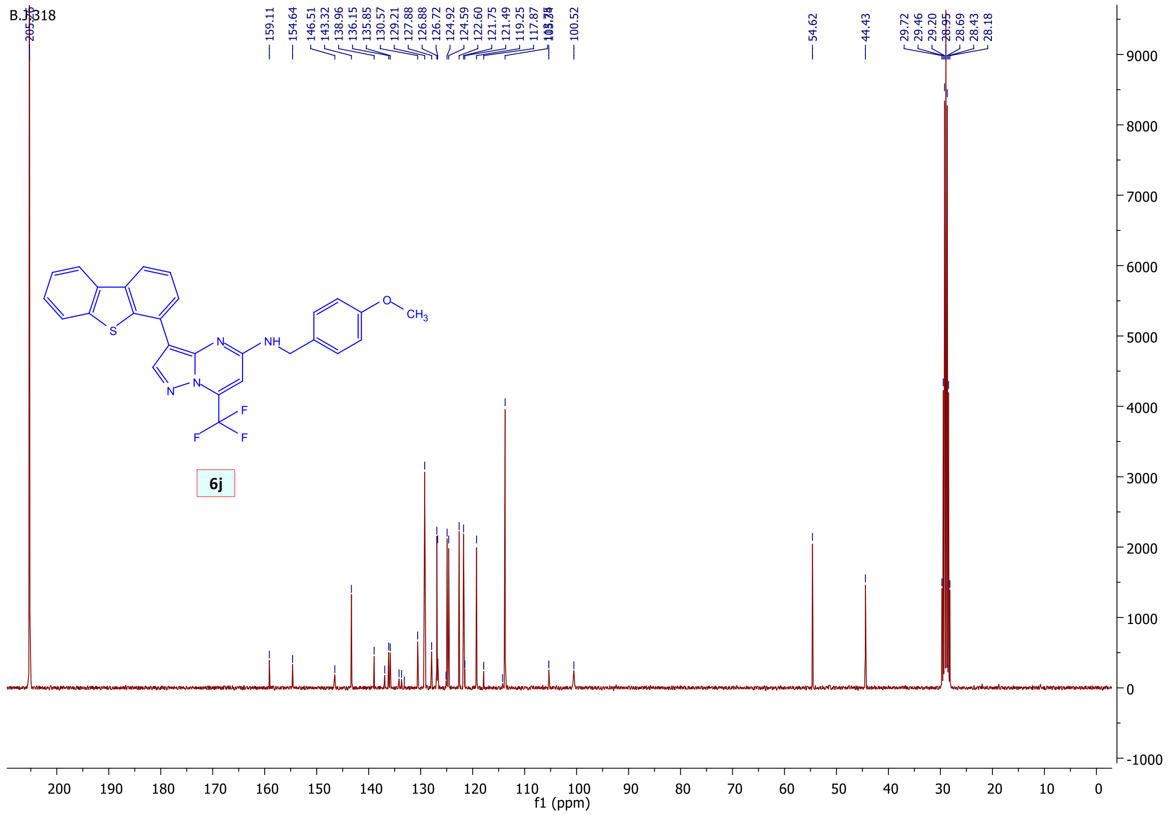

5-N-[4-(hydroxycyclohexyl)amino]-3-(naphthalen-2-yl)-7-(trifluoromethyl)pyrazolo[1,5-a]pyrimidine (**6k**).

^{1}H NMR (300 MHz, Acétone-d_6)

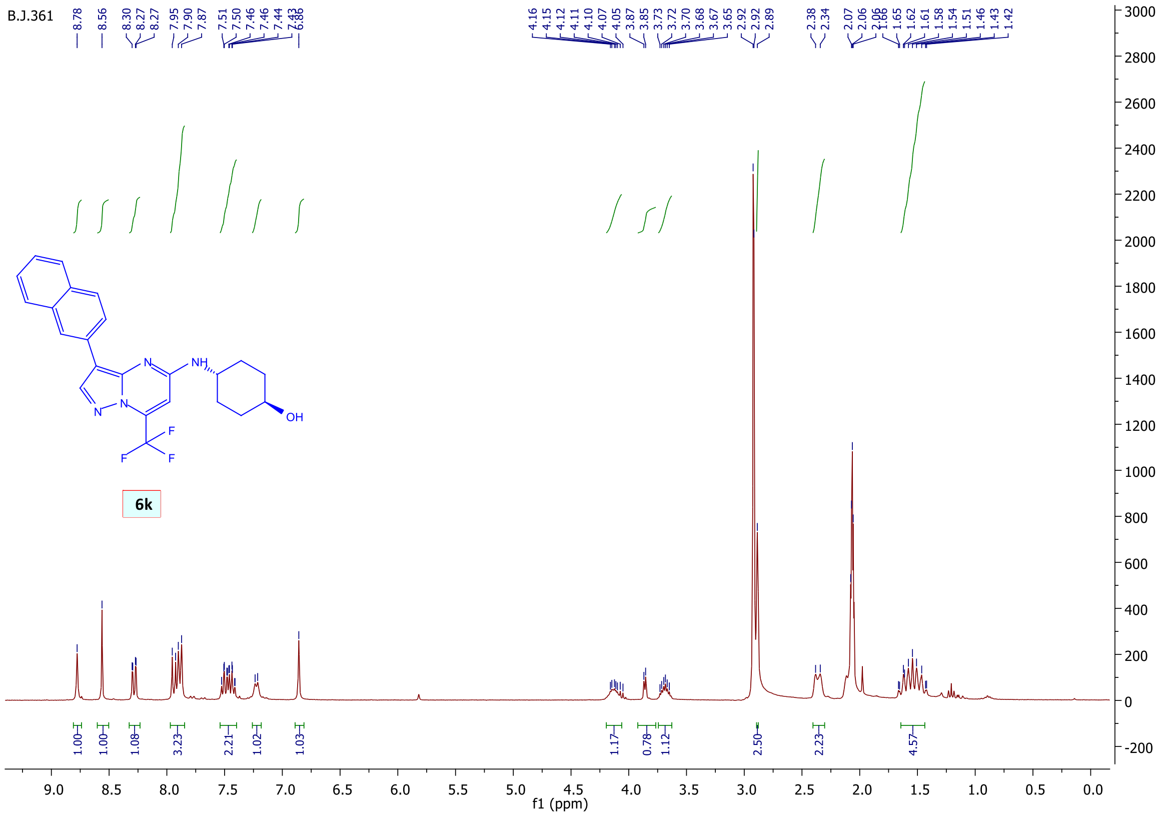

^{19}F NMR (282 MHz, Acétone-d_6)

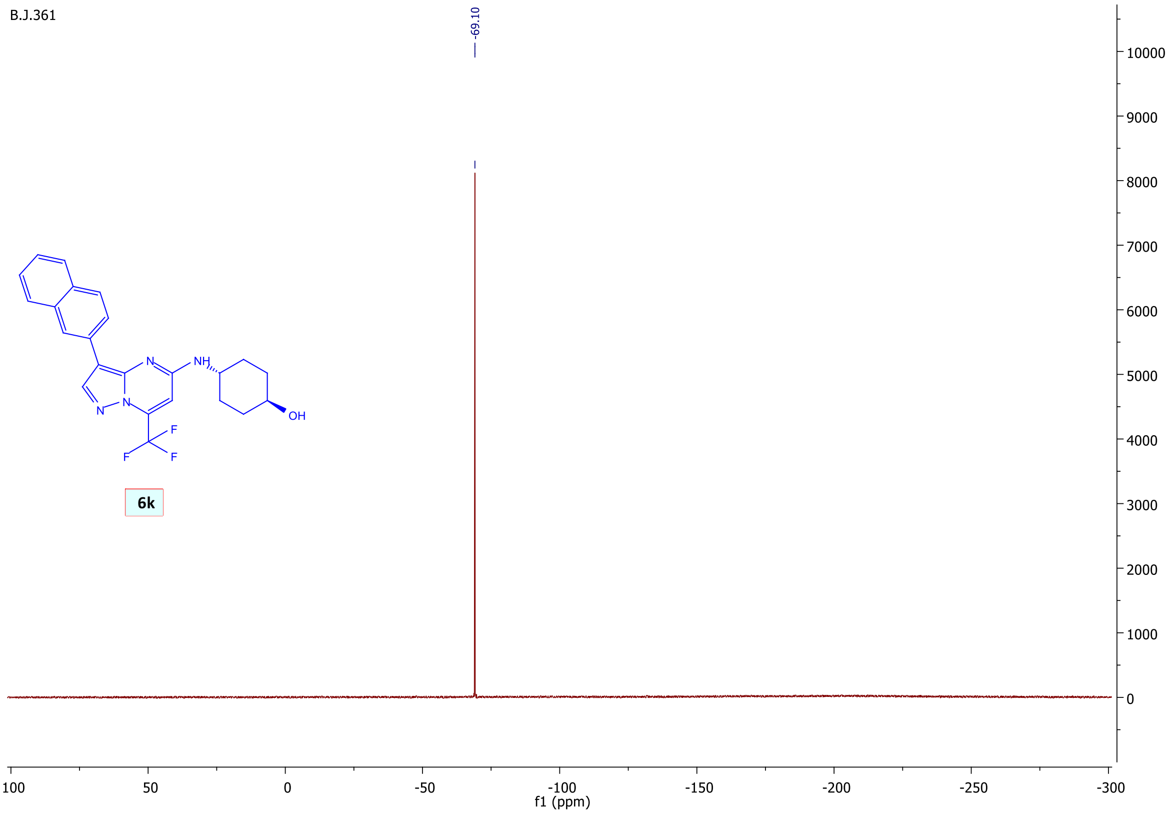

^{13}C NMR (75 MHz, DMSO-d_6)

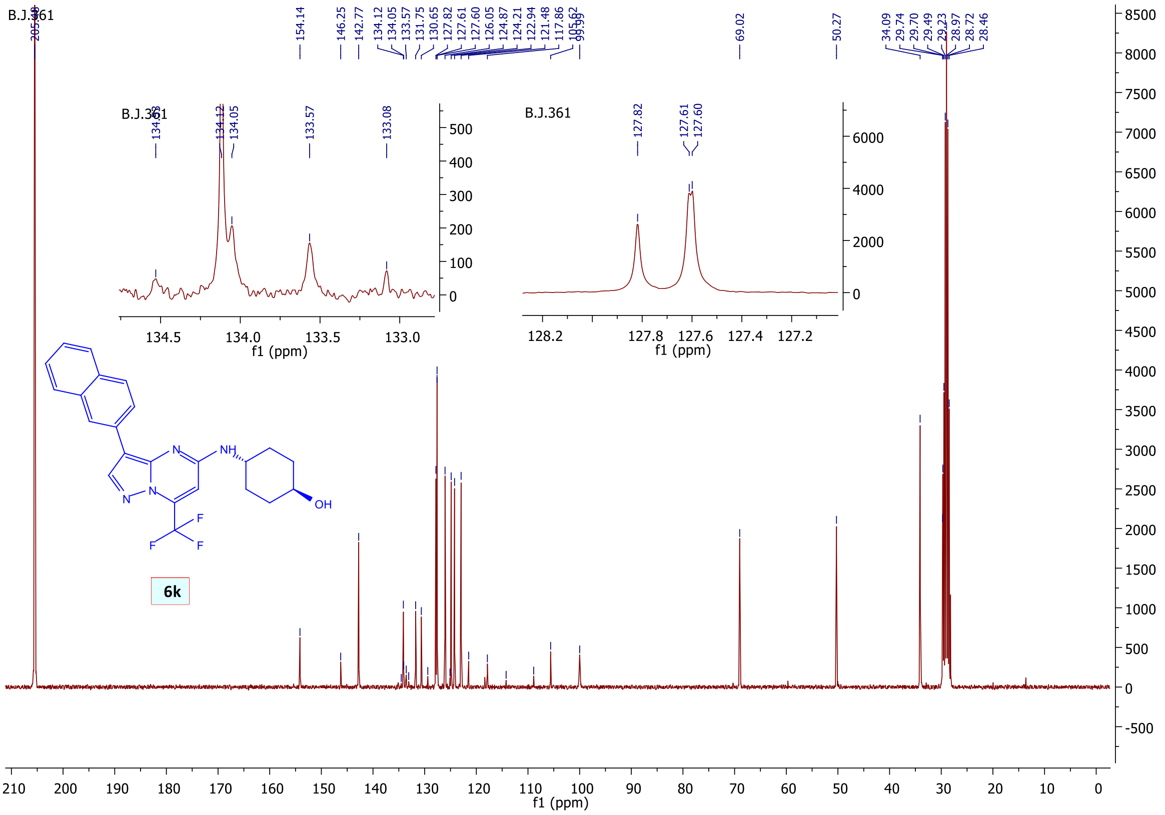

N-[4-(hydroxycyclohexyl)amino]-3-(3-(trifluoromethyl)phenyl)-7-(trifluoromethyl)pyrazolo[1,5-a]pyrimidine (**6l**).

^{1}H NMR (300 MHz, Acétone-d_6)

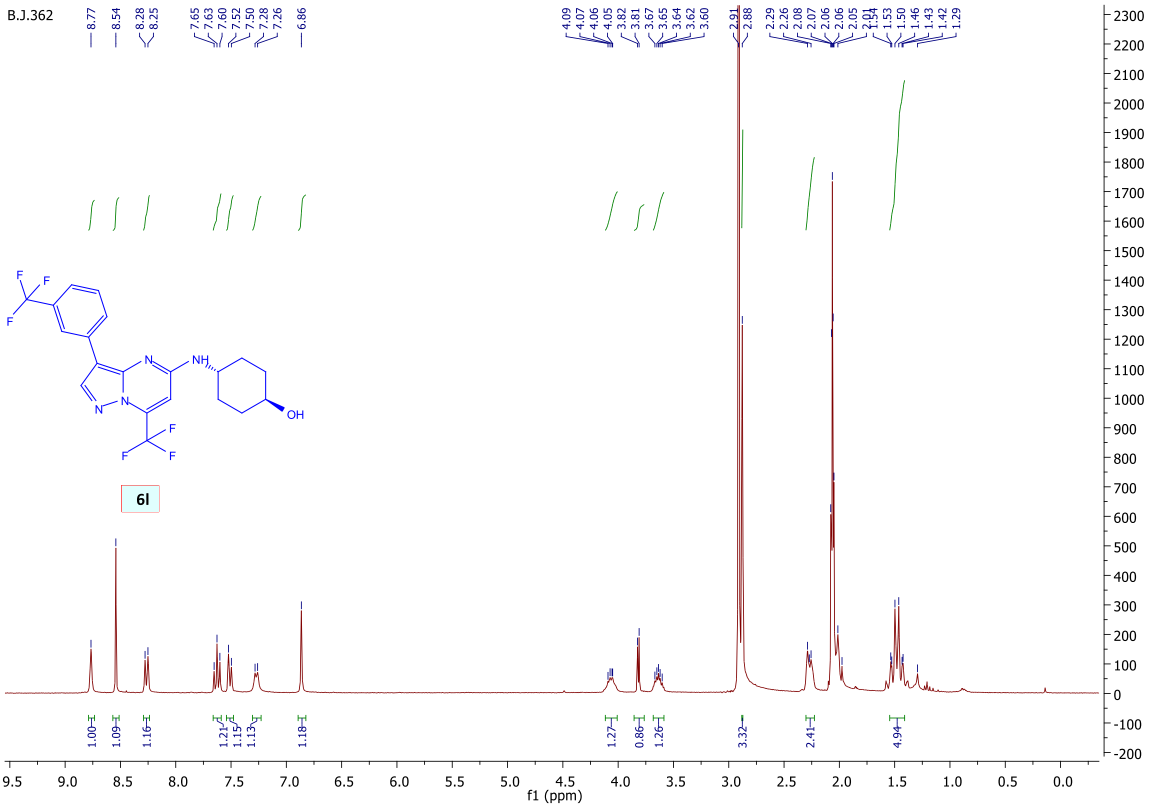

^{19}F NMR (282 MHz, Acétone-d_6)

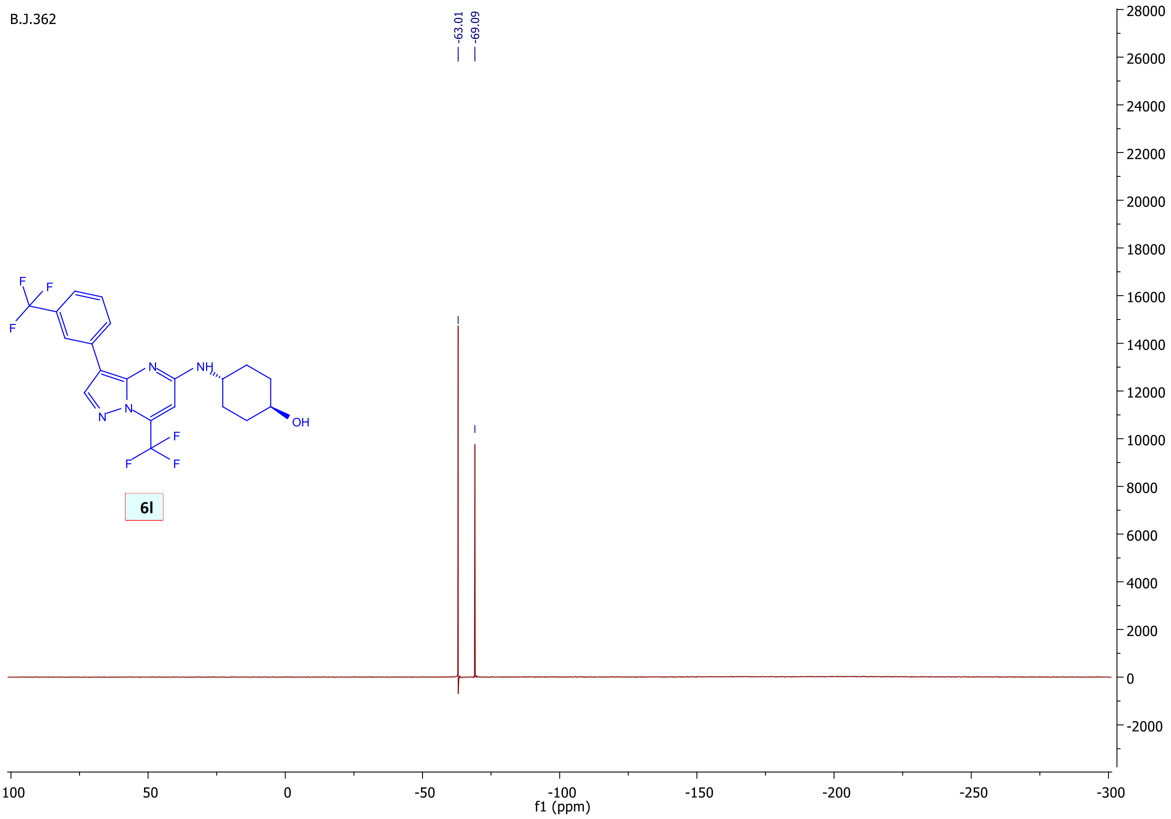

^{13}C NMR (75 MHz, Acétone-d_6)

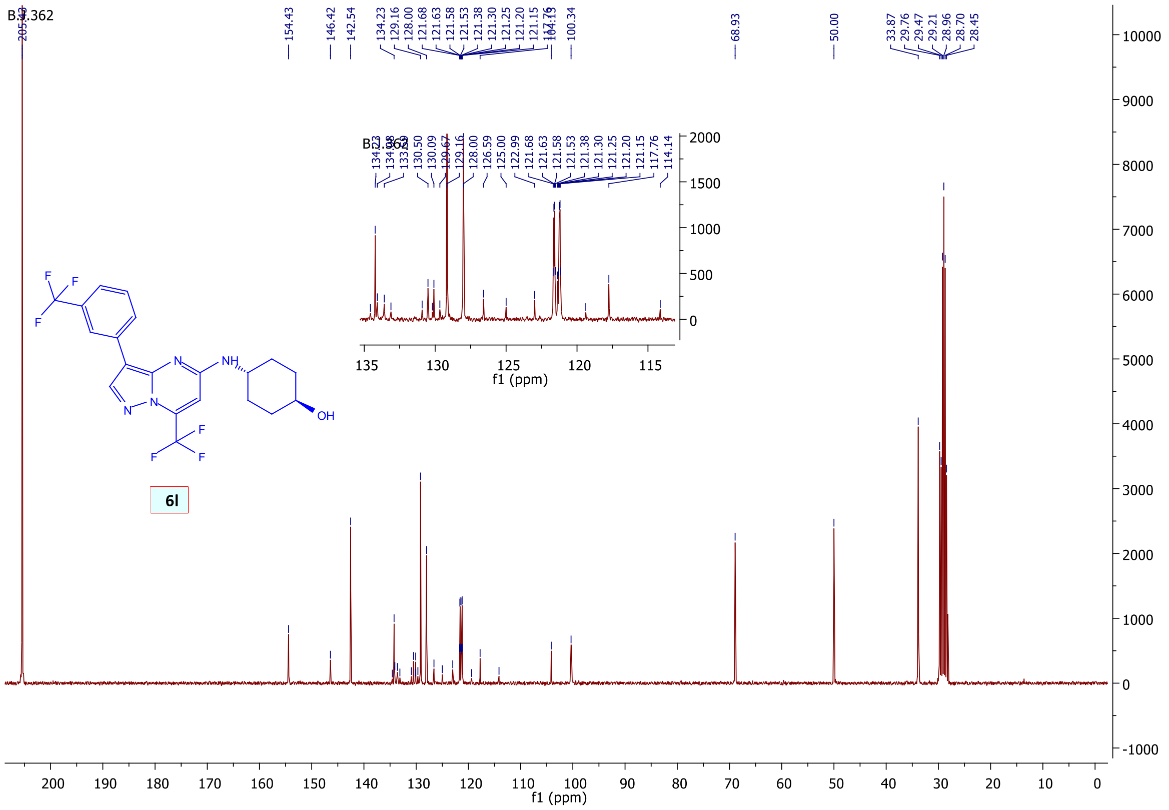

3-(naphthalen-2-yl)-5-[2-(tertiobutoxycarboxylamino)-N-éthylamino]-7-(trifluoromethyl)pyrazolo[1,5-a]pyrimidine (**6m**).

^{1}H NMR (300 MHz, Acétone-*d$_6$*)

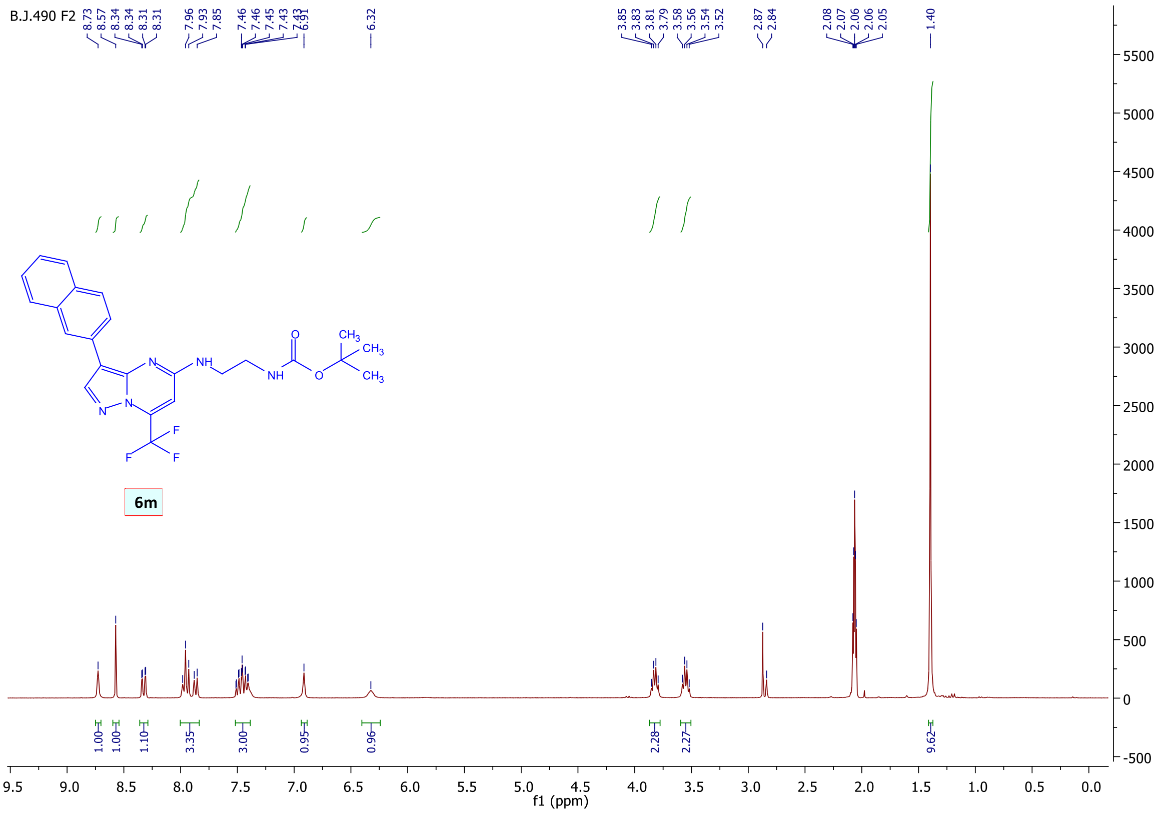

^{19}F NMR (282 MHz, Acétone-*d$_6$*)

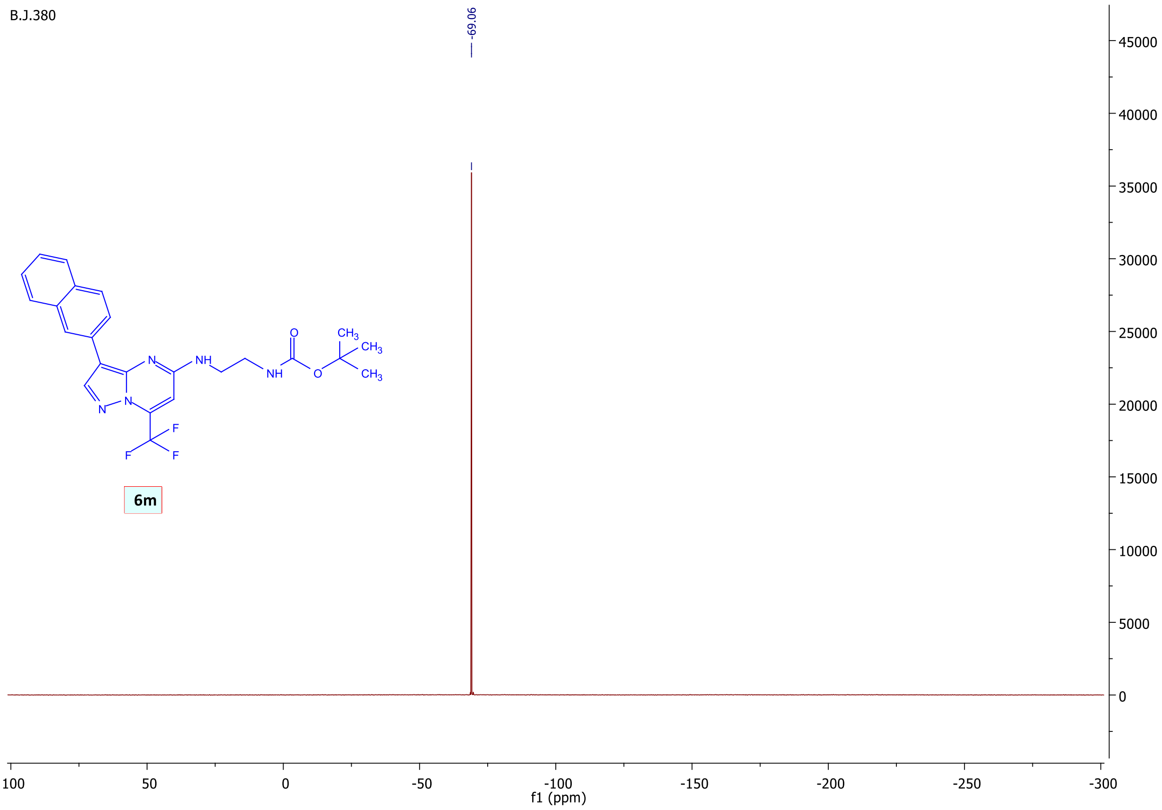

^{13}C NMR (75 MHz, Acétone-d_6)

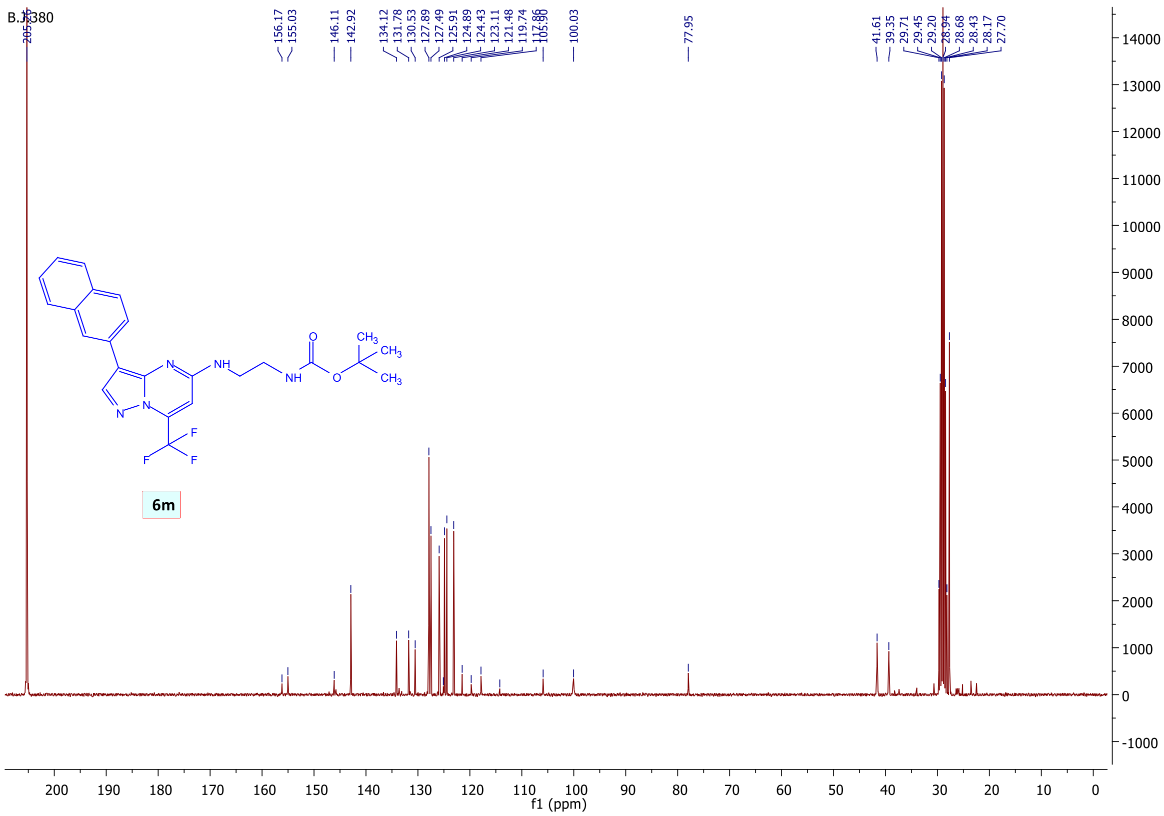

5-[2-amino-N-éthylamino]-3-(Naphthalen-2-yl)-7-(trifluoromethyl)pyrazolo[1,5-a]pyrimidine (**9**)

^{1}H NMR (300 MHz, Acétone-d_6)

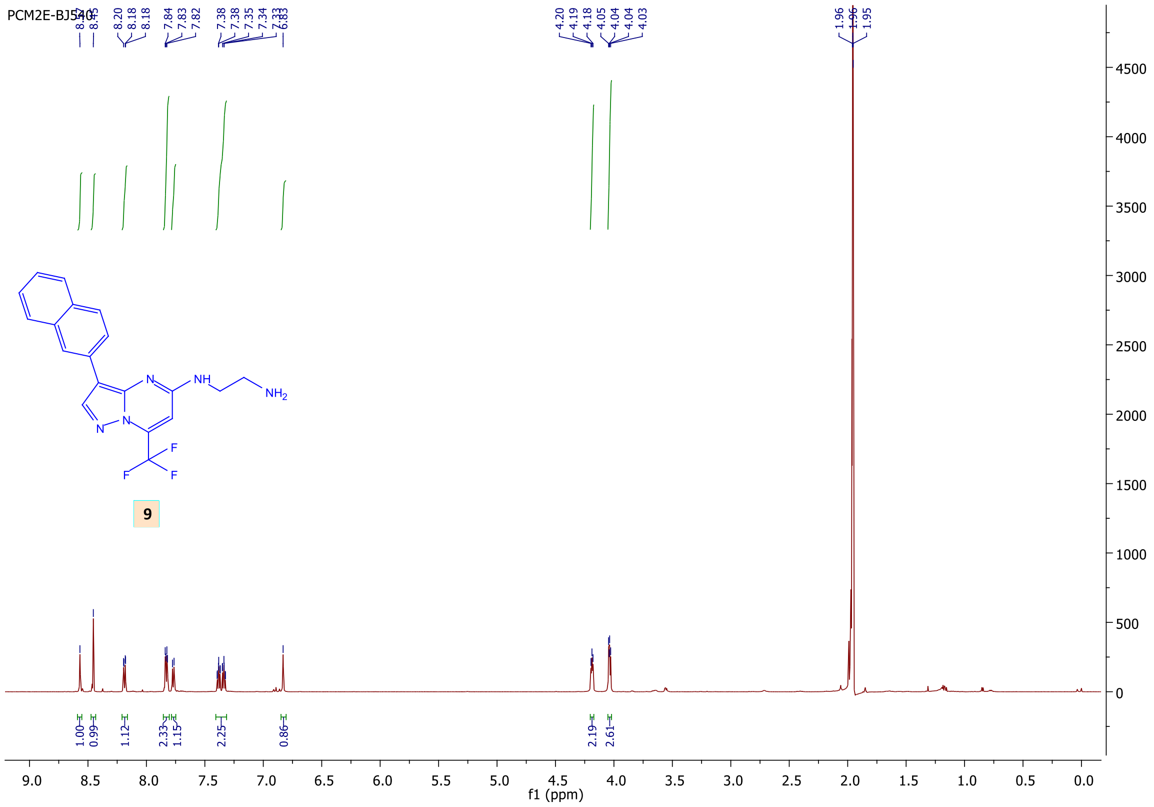

Supplement: Supplementary file 1 [file molecules-25-02062-s001.pdf]
